# Cdt1-binding protein GRWD1 is a novel histone-binding protein that facilitates MCM loading through its influence on chromatin architecture

**DOI:** 10.1093/nar/gkv509

**Published:** 2015-05-18

**Authors:** Nozomi Sugimoto, Kazumitsu Maehara, Kazumasa Yoshida, Shuhei Yasukouchi, Satoko Osano, Shinya Watanabe, Masahiro Aizawa, Takashi Yugawa, Tohru Kiyono, Hitoshi Kurumizaka, Yasuyuki Ohkawa, Masatoshi Fujita

**Affiliations:** 1 Department of Cellular Biochemistry, Graduate School of Pharmaceutical Sciences, Kyushu University, 3-1-1 Maidashi, Higashi-ku, Fukuoka 812-8582, Japan; 2 Faculty of Medicine, Division of Epigenetics, Kyushu University, 3-1-1 Maidashi, Higashi-ku, Fukuoka 812-8582, Japan; 3 Division of Virology, National Cancer Center Research Institute, 5-1-1 Tsukiji, Chuo-ku, Tokyo 104-0045, Japan; 4 Laboratory of Structural Biology, Graduate School of Advanced Science and Engineering, Waseda University, 2–2 Wakamatsu-cho, Shinjuku-ku, Tokyo 162-8480, Japan

## Abstract

Efficient pre-replication complex (pre-RC) formation on chromatin templates is crucial for the maintenance of genome integrity. However, the regulation of chromatin dynamics during this process has remained elusive. We found that a conserved protein, GRWD1 (glutamate-rich WD40 repeat containing 1), binds to two representative replication origins specifically during G1 phase in a CDC6- and Cdt1-dependent manner, and that depletion of GRWD1 reduces loading of MCM but not CDC6 and Cdt1. Furthermore, chromatin immunoprecipitation coupled with high-throughput sequencing (Seq) revealed significant genome-wide co-localization of GRWD1 with CDC6. We found that GRWD1 has histone-binding activity. To investigate the effect of GRWD1 on chromatin architecture, we used formaldehyde-assisted isolation of regulatory elements (FAIRE)-seq or FAIRE-quantitative PCR analyses, and the results suggest that GRWD1 regulates chromatin openness at specific chromatin locations. Taken together, these findings suggest that GRWD1 may be a novel histone-binding protein that regulates chromatin dynamics and MCM loading at replication origins.

## INTRODUCTION

DNA replication is of fundamental importance in cells and is strictly regulated to occur precisely once per cell cycle. An essential step in DNA replication is the formation of a pre-replication complex (pre-RC) by the ORC, CDC6, Cdt1 and MCM helicase complexes at a replication origin during the low Cdk period. After activation of the MCM helicase by Cdk, reassembly of the pre-RC is strictly prohibited to prevent rereplication (1–3). In human cells, Cdt1 strongly promotes MCM loading (4,5), and its activity is tightly restricted by multiple mechanisms (1,6).

Theoretically, loading of two MCM complexes at one replicon would be sufficient for replication. However, it has been demonstrated that excess MCM loading is crucial for maintenance of genome integrity. For example, although cells with depleted MCM replicate at normal rates, they are hypersensitive to replicative stress and defective in Rad17-dependent ATR-mediated replication checkpoint activation (7–9). Moreover, a mutation in MCM4 is viable but causes adenocarcinoma (10). Similarly, mice with reduced expression of MCM2 develop normally but their life spans are greatly reduced (11). These findings suggest that efficient MCM loading is critical for tolerance of replication stress and activation of the checkpoint.

The assembly of chromosomal DNA and histones into nucleosomes is the most fundamental step in eukaryotic chromatin structure. The nucleosome structure generally restricts access by various factors that facilitate a variety of DNA-templated processes. Therefore, the deposition, remodeling and eviction of nucleosomes are important for almost all DNA-related operations. In transcription, chromatin-remodeling proteins, histone chaperones and histone-modifying enzymes are thought to synergistically stimulate the reaction. The situation may be similar for efficient pre-RC formation on chromatin. In this regard, it has been reported that HBO1 enhances licensing through its acetylation activity (12–14). In addition, we demonstrated that the chromatin remodeler SNF2H promotes MCM loading by binding to Cdt1 (6,15). Histone chaperones bind histones and play crucial roles in mediating nucleosome assembly/disassembly (16,17). In the case of pre-RC formation, however, the histone chaperones involved remain elusive.

We previously identified GRWD1 (glutamate-rich WD40 repeat containing 1) as a novel Cdt1-binding protein (6). GRWD1 is highly conserved throughout eukaryotes (18). Rrb1, the budding yeast homolog of GRWD1, is essential for growth, is involved in early ribosome assembly and genetically interacts with Orc6 (19–21). In addition, Rrb1 interacts with Yph1, which functions cooperatively with the Origin Recognition Complex (ORC) and Mini Chromosome Maintenance (MCM) (22). However, in metazoan cells, the function of GRWD1 is mostly unknown, except for a possible involvement in ribosome biogenesis (18) and identification as a candidate substrate-receptor of Cul4-DDB1 ubiquitin ligase (23,24). Here, we show that GRWD1 is a histone-binding protein regulating chromatin dynamics and MCM loading.

## MATERIALS AND METHODS

### ChIP-seq and data analysis

Preparation of cross-linked chromatin was carried out essentially as described for ChIP-qPCR. However, MNase treatment (New England Biolabs, 500 U/200μl lysate, 37°C for 30 min) was added after sonication. The ChIP library was prepared with the Illumina protocol and sequencing analysis was performed using the Genome Analyzer GAIIx (Illumina KK). Sequencing for the MCM7 and HA-GRWD1 ChIP analysis was performed using the HiSeq1500 (Illumina KK). The sequence reads were aligned to the human genome (hg19) using Bowtie software (version 0.12.8; parameter -v3 -m1). Peak detection and identification of the binding sites were obtained by correcting from Input DNA, using MACS2 broad peak detection. Genomic annotations including transcription start sites (TSSs) and transcription end sites (TESs) were determined using RefSeq. Co-localization analyses of ChIP-Seq data were obtained as described previously (25,26).

To estimate the ChIP-seq signals, we utilized the moving-average method in which the number of mapped reads was calculated every 0.1 kb interval with 1 kb window size. In addition, read numbers of the ChIP samples were normalized using Reads Per Million (RPM) (27), and further divided by the RPM of the input samples.

### FAIRE

HeLa cells were cross-linked with 1% formaldehyde for 5 min at room temperature. Glycine was added to a final concentration of 125 mM, and the cells were rinsed with cold PBS and harvested. Cells were then lysed with 2% TX-100, 1% SDS, 100 mM NaCl, 1 mM EDTA and 10 mM Tris-HCl (pH 8.0) containing a protease inhibitor cocktail and sonicated for 18 min (30 s on/60 s off cycles) using a Bioruptor (Diagenode) set at the highest intensity. Samples were centrifuged at 15 000 rpm for 10 min at 4°C to precipitate cellular debris. For input control samples, prior to phenol-chloroform extractions, the soluble chromatin was treated with proteinase K and incubated for 6 h at 65°C to reverse cross-linking. Chromatin samples were then subjected to two consecutive phenol-chloroform extractions. FAIRE chromatin samples recovered in the aqueous layers were then subjected to reversion of cross-linking. Finally, the DNAs were ethanol precipitated. Input and FAIRE DNAs were then analyzed by agarose gel electrophoresis followed by EtBr staining.

For FAIRE-Seq, the DNAs were subjected to Seq (HiSeq1500, Illumina KK) and data analysis was performed essentially as described above. Read numbers of the FAIRE samples were normalized by subtracting those of the input samples. FAIRE-qPCR was performed on the input and FAIRE DNAs essentially as described for ChIP-qPCR.

## RESULTS

### GRWD1 is present in the nucleus throughout interphase and is a chromatin/nuclear matrix-binding protein

**Figure 1.**
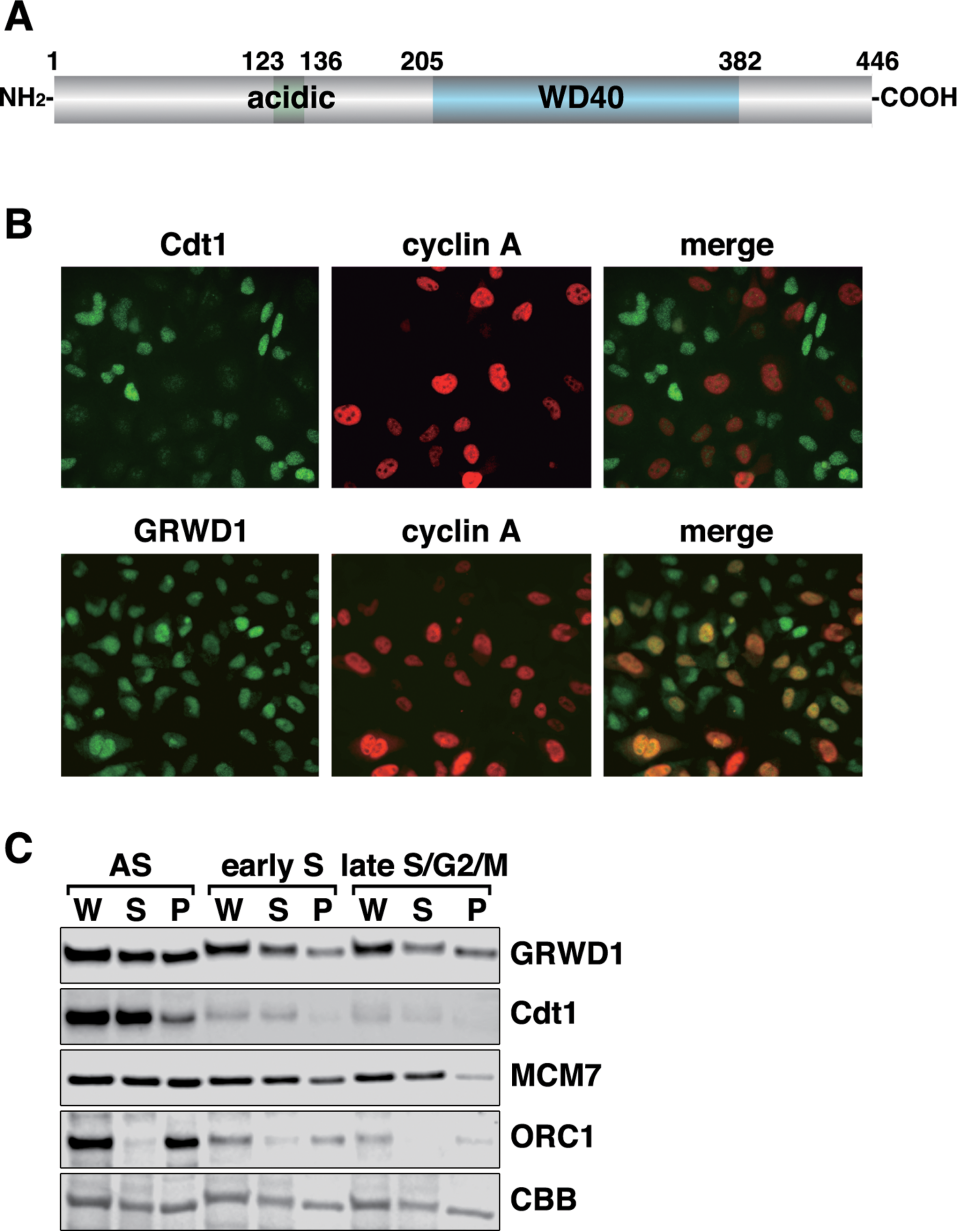
GRWD1 is present in the nucleus throughout interphase and is a chromatin/nuclear matrix-binding protein. (**A**) Schematic representation of GRWD1 domain structure. An acidic domain and five WD40 repeats are indicated. (**B**) HeLa cells were double-immunostained with anti-cyclin A and anti-GRWD1 or anti-cyclin A and anti-Cdt1 antibodies. (**C**) HeLa cells were allowed to grow asynchronously (AS), treated with hydroxyurea (HU) and then released for 2 h (for early S phase) or 9 h (for late S/G2/M phase), and then subjected to chromatin-binding assays. Coomassie Brilliant Blue (CBB) staining served as the loading control. W, whole cell extract; S, Triton X-100-extractable soluble fraction; and P, chromatin/nuclear matrix-bound fraction.

GRWD1 is composed of 446 amino acids, including a glutamate-rich acidic domain and a WD40 repeat domain (Figure 1A). A similar acidic domain has been found in many proteins involved in histone binding, and the WD40 repeat domain of GRWD1 has homology with the p48 subunit of CAF1, which can interact with histones (17).

We raised rabbit anti-human GRWD1 antibodies, which recognized a ∼55 kDa band in immunoblots (Supplementary Figure S1A) (6) and yielded nuclear signals in immunostaining (Supplementary Figure S1B). These signals were diminished when GRWD1 was silenced using siRNAs (Supplementary Figure S1A and B). Interphase nuclear GRWD1 staining was observed both in cyclin A-negative, G1-phase cells and in cyclin A-positive, S-phase and G2-phase cells (Figure 1B). We also carried out chromatin-binding assays in HeLa cells synchronized in early S phase or late S/G2/M phase as well as in asynchronous cells (Figure 1C). Cdt1 and ORC1 protein levels decreased during S phases, whereas MCM7 dissociated from chromatin in late S/G2/M phase, as expected (1). In all situations tested, about half of the GRWD1 protein was detected in chromatin/nuclear matrix-bound fractions. Thus, GRWD1 is present in nuclei throughout interphase, and is a chromatin/nuclear matrix-bound protein. As shown below, GRWD1 is detached from chromatin in quiescent cells (Supplementary Figure S3D), suggesting that it is involved in cell growth control. We also found that GRWD1 is detached from chromatin at the onset of mitosis and rebinds at telophase when the pre-RC is formed (Supplementary Figure S1D and E).

### GRWD1 physically interacts with CDC6 and Cdt1

**Figure 2.**
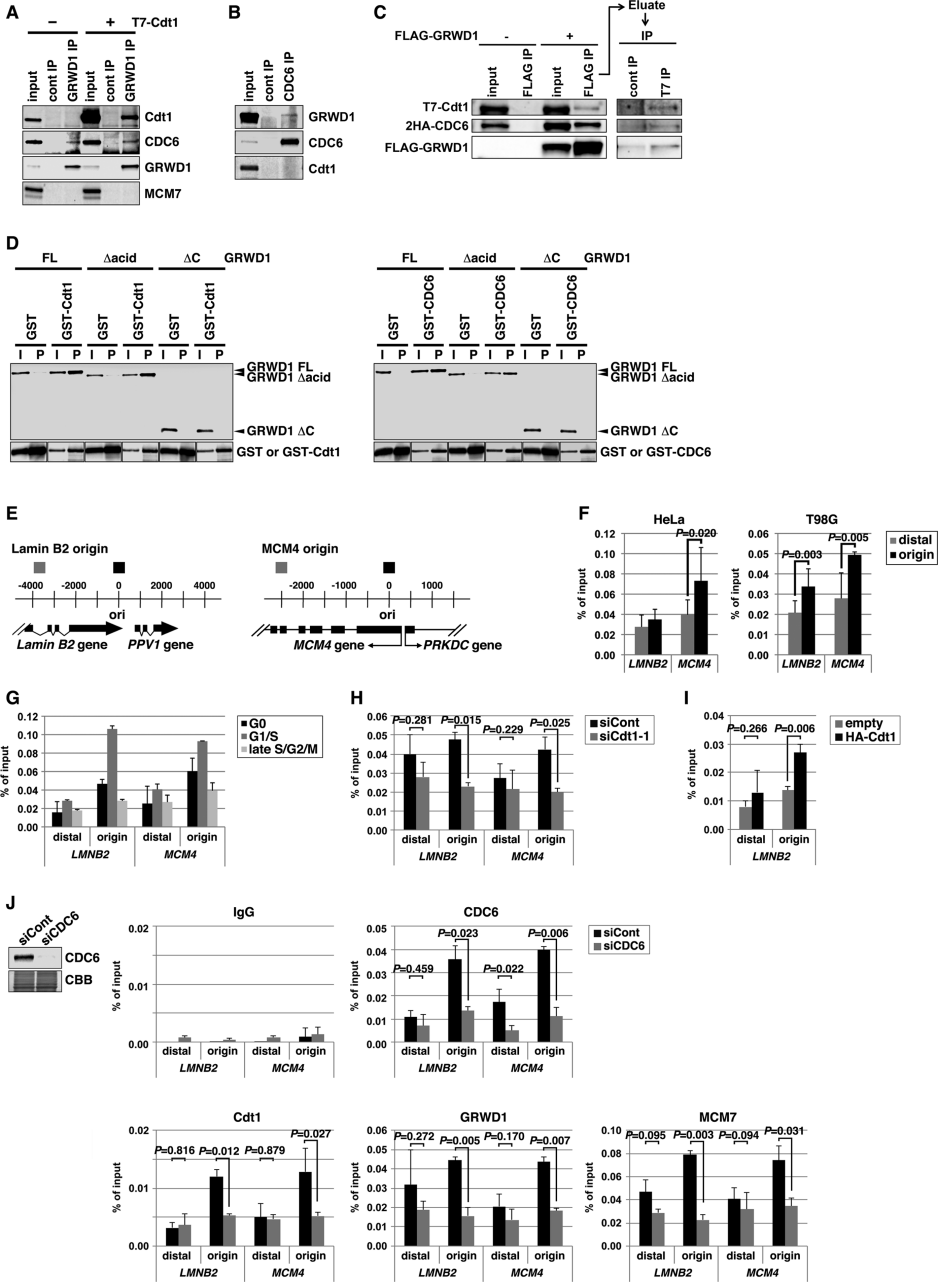
GRWD1 interacts with Cdt1 and CDC6 and binds specifically to replication origins at the *lamin B2* and *MCM4* loci during G1 phase in a CDC6- and Cdt1-dependent manner. (**A**) HEK293T cells transfected with empty or T7-Cdt1 expression vectors were immunoprecipitated with anti-GRWD1 antibody or control rabbit IgG. The precipitates (IP) were then immunoblotted with the indicated antibodies. Two percent of the input was also loaded. (**B**) Nuclear extracts prepared from HEK293T cells were immunoprecipitated with anti-CDC6 antibody. Immunoprecipitates were subjected to immunoblotting with the indicated antibodies. Two percent of the input was also loaded. (**C**) Soluble extracts from HEK293T cells transfected with the indicated expression vectors were immunoprecipitated with anti-Flag antibody. The first immunoprecipitates were eluted with Flag peptide and then re-immunoprecipitated with anti-T7 antibody. Each precipitate (IP) was then immunoblotted with the indicated antibodies. Fifteen percent of the input for first IP and 10% of the input (i.e. the first precipitates) for second IP were also loaded. (**D**) GST-Cdt1, GST-CDC6 or GST was incubated with bacterially produced full length GRWD1 (FL) or its truncated mutants (Δacid, lacking the acidic domain; ΔC, lacking C-terminal amino acids 207–446). GST and the associated proteins were then collected on glutathione beads and subjected to immunoblotting with anti-GRWD1 (upper panel) or anti-GST antibody (lower panel). Fifteen percent of the input sample (I) was analyzed with the precipitate (P). (**E**) Schematic diagram of the origins at the human *lamin B2* and *MCM4* loci. Positions of qPCR primer pairs used to detect the origins and distal sequences are shown above (black and gray boxes, respectively). Coordinates flanking the origin are given in base pairs. (**F**) ChIP-qPCR assay with anti-GRWD1 antibody was performed using a previously described set of chromatin samples from asynchronously growing HeLa or T98G cells (15). Results are shown as the percent of input DNA. The means ± SD are shown (HeLa, *n* = 8; T98G, *n* = 6). Control IgG gave signals below 0.005% of the inputs (15). (**G**) ChIP-qPCR assay with anti-GRWD1 antibody was performed using a previously described set of chromatin samples from T98G cells synchronized at the G0, G1/S and late S/G2/M phases (15). The means ± SD are shown (*n* = 3). (**H**) ChIP-qPCR assay with anti-GRWD1 antibody was performed using a previously described set of chromatin samples from HeLa cells transfected with control or Cdt1 (siCdt1–1) siRNAs for 48 h (15). Depletion of Cdt1 by specific siRNA, and a corresponding reduction in origin enrichment with anti-Cdt1 and anti-MCM7 antibodies, has already been confirmed (15). The means ± SD are shown (*n* = 3). (**I**) ChIP-qPCR assay with anti-GRWD1 antibody was performed for a previously described set of chromatin samples from HEK293T cells transfected with the indicated expression vectors for 48 h (15). The means ± SD are shown (*n* = 3). (**J**) HeLa cells transfected with control or CDC6 (mixture of siCDC6–1&3) siRNAs for 48 h were subjected to immunoblotting and ChIP-qPCR assay with the indicated antibodies. For ChIP data, the means ± SD are shown (*n* = 3).

We previously showed that GRWD1 binds to GST-Cdt1 in pull-down assays and can be co-immunoprecipitated with overexpressed T7-Cdt1 (6). Here, we further show that ectopically overexpressed T7-Cdt1 reciprocally co-immunoprecipitated with endogenous GRWD1 (Figure 2A). Endogenous CDC6 co-immunoprecipitated with endogenous GRWD1 (Figure 2A), and *vice versa* (Figure 2B). In these assays, coprecipitation of endogenous Cdt1 could not be observed. When overexpressed, the ternary complexes of GRWD1, CDC6 and Cdt1 were detectable (Figure 2C). In pull-down assays, purified GRWD1 bound directly to purified GST-Cdt1 and GST-CDC6, respectively (Figure 2D). C-terminal amino acids 207–446 containing the WD40 repeats, but not the acidic domain, were required for binding (Figure 2D).

### GRWD1 binds to replication origins at the *lamin B2* and *MCM4* loci in a cell cycle-regulated manner

We examined whether GRWD1 is present at two well-known replication origins near the *lamin B2* (*LMNB2*) and *MCM4* genes. We performed chromatin immunoprecipitation (ChIP) with anti-GRWD1 antibodies on asynchronously growing cells, and assessed the presence of origin sequences by quantitative real-time PCR analysis (qPCR) (Figure 2E). We previously reported that Cdt1-, MCM7- or MCM3-specific antibodies efficiently immunoprecipitated DNA sequences included in the replication origin regions of both loci in two different human cell lines, HeLa and T98G (15). Here, we used the same set of chromatin samples and showed that anti-GRWD1 antibodies also efficiently enriched the origin sequences (Figure 2F). Some enrichment of the origin-distal regions was also observed, but less efficient. Control IgG gave signals below 0.005% of the inputs (15). In addition, only a little enrichment of the more distal region (∼10 kb apart) from the *lamin B2* origin was observed with anti-MCM7, -Cdt1 or -GRWD1 antibodies (Supplementary Figure S2A and B). Importantly, silencing of GRWD1 decreased the ChIP signals (see below), confirming the validity of the assay. These data show that GRWD1 indeed binds to replication origins.

We investigated the cell-cycle dependence of GRWD1 binding using previously described chromatin samples from synchronized T98G cells (15). The origin binding was decreased in G0 or late S/G2/M-phase cells, but was significantly elevated in G1/S phase (Figure 2G), reminiscent of the binding profile of pre-RC (15). These results indicate that GRWD1 is associated with origins during G1 phase.

### Origin binding of GRWD1 is dependent on Cdt1 and CDC6

We next examined the dependence of GRWD1 origin loading on Cdt1 and CDC6. We analyzed chromatin from Cdt1 siRNA-treated HeLa cells (15) and found that GRWD1 binding at origins was significantly decreased by Cdt1 silencing (Figure 2H). On the other hand, its binding to the origin-distal regions was not statistically significantly changed by Cdt1 silencing (Figure 2H). Similar results were obtained using another Cdt1 siRNA (Supplementary Figure S2C and D). As shown in Figure 2I, we also found that GRWD1 binding at the *LMNB2* origin was significantly enhanced upon Cdt1 overexpression in HEK293T cells (15). Therefore, recruitment of GRWD1 to origins may be dependent on Cdt1.

We also investigated the effect of CDC6 depletion in HeLa cells. Depletion of CDC6 and reduction in origin enrichment with anti-CDC6 antibody was confirmed by immunoblotting and ChIP-qPCR (Figure 2J). As is the case for Cdt1 silencing (15), the cell-cycle profile was not remarkably affected by CDC6 silencing at this level (28), probably due to the relatively high basal level of expression in HeLa cells. Similarly, silencing of MCM5 does not inhibit normal replication (7). As expected, MCM7 loading was significantly inhibited by CDC6 silencing (Figure 2J). GRWD1 binding at origins was also decreased by CDC6 silencing while its binding to the origin-distal regions was not statistically significantly changed (Figure 2J), suggesting that GRWD1 recruitment might depend on CDC6. However, because Cdt1 loading was inhibited by CDC6 silencing (Figure 2J), we cannot exclude the possibility that this inhibition is mediated by Cdt1 downregulation. Collectively, these results suggest that GRWD1 is recruited onto replication origins via physical interaction with CDC6 and Cdt1. However, the possibility also remains that GRWD1 is recruited to open chromatin regions passively rather than specifically through its interaction with CDC6 and Cdt1. For example, manipulation of CDC6 or Cdt1 expression could primarily change chromatin openness by affecting HBO1 and SNF2H recruitment, and then such change of chromatin openness might influence GRWD1 recruitment. This issue should be addressed in future.

### Silencing of GRWD1 suppresses loading of MCM, but not CDC6 and Cdt1, and perturbs DNA replication while GRWD1 overexpression alleviates MCM loading defect by Cdt1 silencing

**Figure 3.**
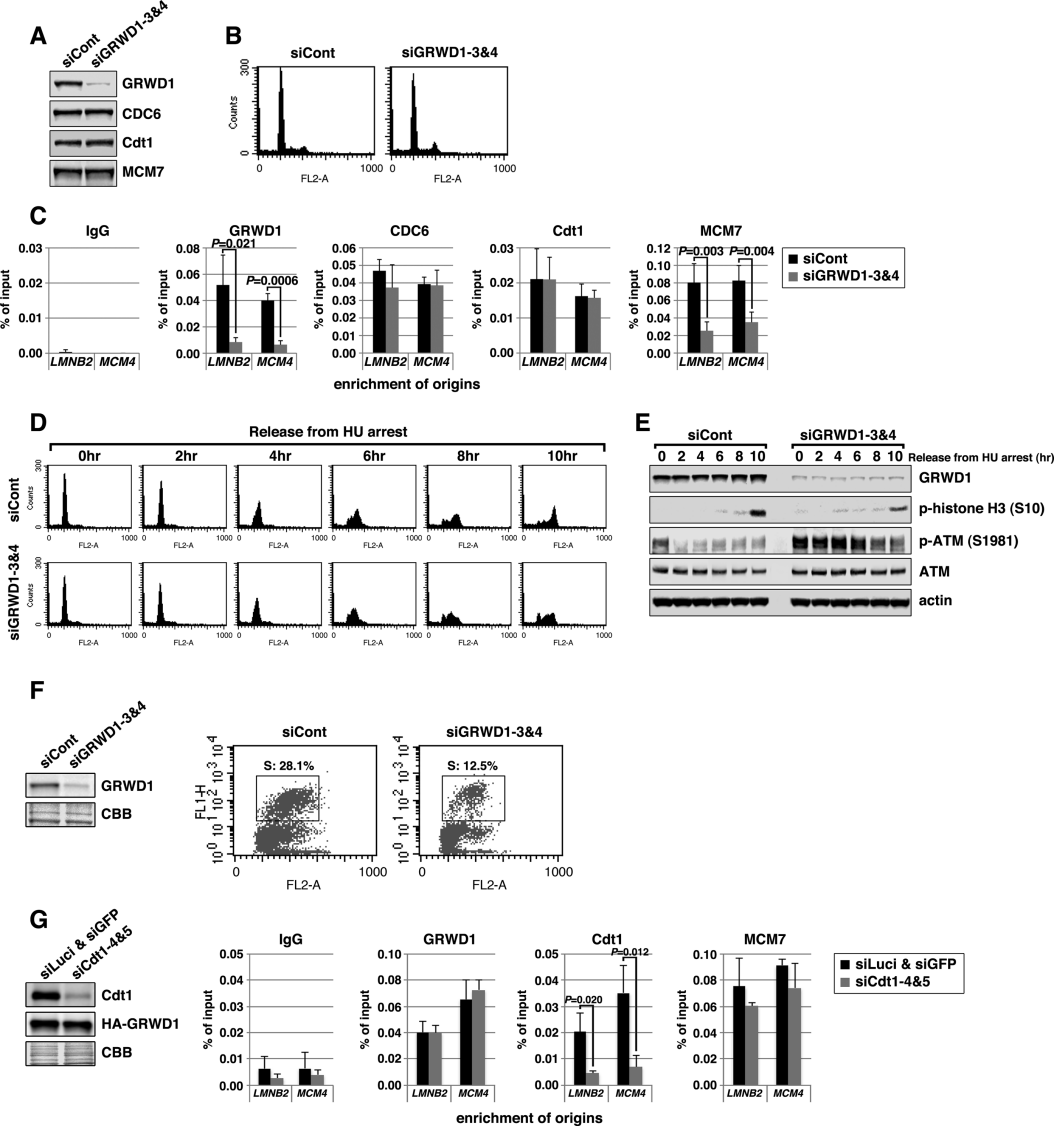
Silencing of GRWD1 suppresses MCM loading and S-phase progression and activates the ATM checkpoint while GRWD1 overexpression alleviates MCM loading defect by Cdt1 inhibition. (**A–C**) HeLa cells transfected with control or GRWD1 (siGRWD1–3&4) siRNAs for 48 h were subjected to immunoblotting (A), FACS (B) and ChIP-qPCR (C). For ChIP data, the means ± SD are shown (*n* = 4). (**D** and **E**) HeLa cells transfected with the indicated siRNAs for 24 h were arrested in S phase with HU and released. The cells were then harvested at the indicated time points and analyzed by FACS (D) and immunoblotting with indicated antibodies (E). Ser10-phosphorylated-histone H3 was used as a mitotic marker. (**F**) HFF2/T cells transfected with control (mixture of siLuci&siGFP) or GRWD1 (mixture of siGRWD1–3&4) siRNAs for 48 h were subjected immunoblotting (left panel) or further labeled with 30 μM BrdU for 30 min. The cells were double stained with anti-BrdU antibody and PI and analyzed by FACS (right panels). (**G**) HeLa cells overexpressing HA-GRWD1 were established by retroviral infection. Cells were then transfected with control (mixture of siLuci&siGFP) or Cdt1 (mixture of siCdt1–4&5) siRNAs for 48 h and then subjected to immunoblotting and ChIP-qPCR assay with the indicated antibodies. For ChIP data, the means ± SD are shown (*n* = 3).

We investigated the role of GRWD1 in MCM loading by siRNA-mediated depletion. Depletion of GRWD1 in asynchronous HeLa cells was confirmed by immunoblotting (Figure 3A and Supplementary Figure S3A). It has been reported that GRWD1 might be involved in ribosome biogenesis, and its depletion leads to a 20% reduction in total protein synthesis (18). However, protein levels of CDC6, Cdt1 and MCM7 were not significantly altered by GRWD1 depletion (Figure 3A; see also Supplementary Figure S3D and E). Cell-cycle profile was slightly affected by GRWD1 depletion, with slight increases in G2/M-phase cells (Figure 3B and Supplementary Figure S3B). The increase in G2/M-phase cells suggests that GRWD1 might be also involved in mitotic progression. On the other hand, S phase population was not remarkably decreased, as is the case for silencing of Cdt1, CDC6 or MCM5 (7,15,28). This may be due to the relatively high basal expression levels of the initiation proteins (29–31) and GRWD1 (Supplementary Figure S1C) in cancer-derived HeLa cells. Next, we subjected chromatin from GRWD1-depleted cells to ChIP-qPCR (Figure 3C). As expected, enrichment of origins by anti-GRWD1 antibody was reduced by GRWD1 knockdown. CDC6 and Cdt1 binding at origins was not changed. By contrast, MCM7 binding at origins was significantly suppressed. Similar results were observed with a second siRNA targeting GRWD1 (Supplementary Figure S3A–C). The inhibitory effect of GRWD1 silencing on MCM loading was also observed in chromatin-binding assays using T98G cells (Supplementary Figure S3D–G). These data suggest that GRWD1 association with origins occurs after CDC6 and Cdt1 loading, to promote loading of the MCM.

We further examined the effects of GRWD1 depletion on DNA replication. HeLa cells were transfected with siRNAs, arrested in early S phase by hydroxyurea treatment, and then released. GRWD1 depletion slightly slowed S-phase progression and delayed entry into M phase (Figure 3D, E and Supplementary Figure S3H). Moderate inhibition of DNA replication by GRWD1 depletion was also observed in asynchronous untransformed fibroblasts (Figure 3F). The relatively moderate effect of GRWD1 depletion on replication might be due to the fact that excess MCM is not necessarily required for normal replication, as mentioned above. Interestingly, GRWD1 depletion moderately activated the ATM DNA-damage checkpoint kinase in cells treated with hydroxyurea and released (Figure 3E and Supplementary Figure S3H). Moderate ATM activation was also observed in GRWD1-depleted asynchronous cells without drug treatment (Supplementary Figure S3I). These data suggest its importance in the maintenance of genome integrity. The phenotypes observed by GRWD1 depletion are partly similar to those by MCM5 depletion (7). Thus, slowdown of S-phase progression and DNA-damage induction in GRWD1-depleted cells could be attributed, at least in part, to insufficient MCM loading. However, as GRWD1 may also function in other processes (see below), perturbation of these processes would also affect the integrity of chromatin and cell cycle progression.

As we have shown previously (15) and above (Figure 2H and Supplementary Figure S2C and D), Cdt1 depletion leads to decrease in GRWD1 and MCM7 binding at origins. To examine whether GRWD1 directly supports the Cdt1 function in MCM loading at origins, we prepared HeLa cells overexpressing HA-GRWD1 and investigated the effect of GRWD1 overexpression on Cdt1 depletion. Similar to the parental HeLa cells ((15); Figure 2H and Supplementary Figure S2C and D), Cdt1 binding at origins was significantly decreased by Cdt1 silencing (Figure 3G). Importantly, GRWD1 and MCM7 binding at origins was not reduced by Cdt1 silencing in HA-GRWD1-overexpressed cells (Figure 3G), further arguing direct involvement of GRWD1 in Cdt1-dependent MCM loading.

### GRWD1 has a histone-binding activity

**Figure 4.**
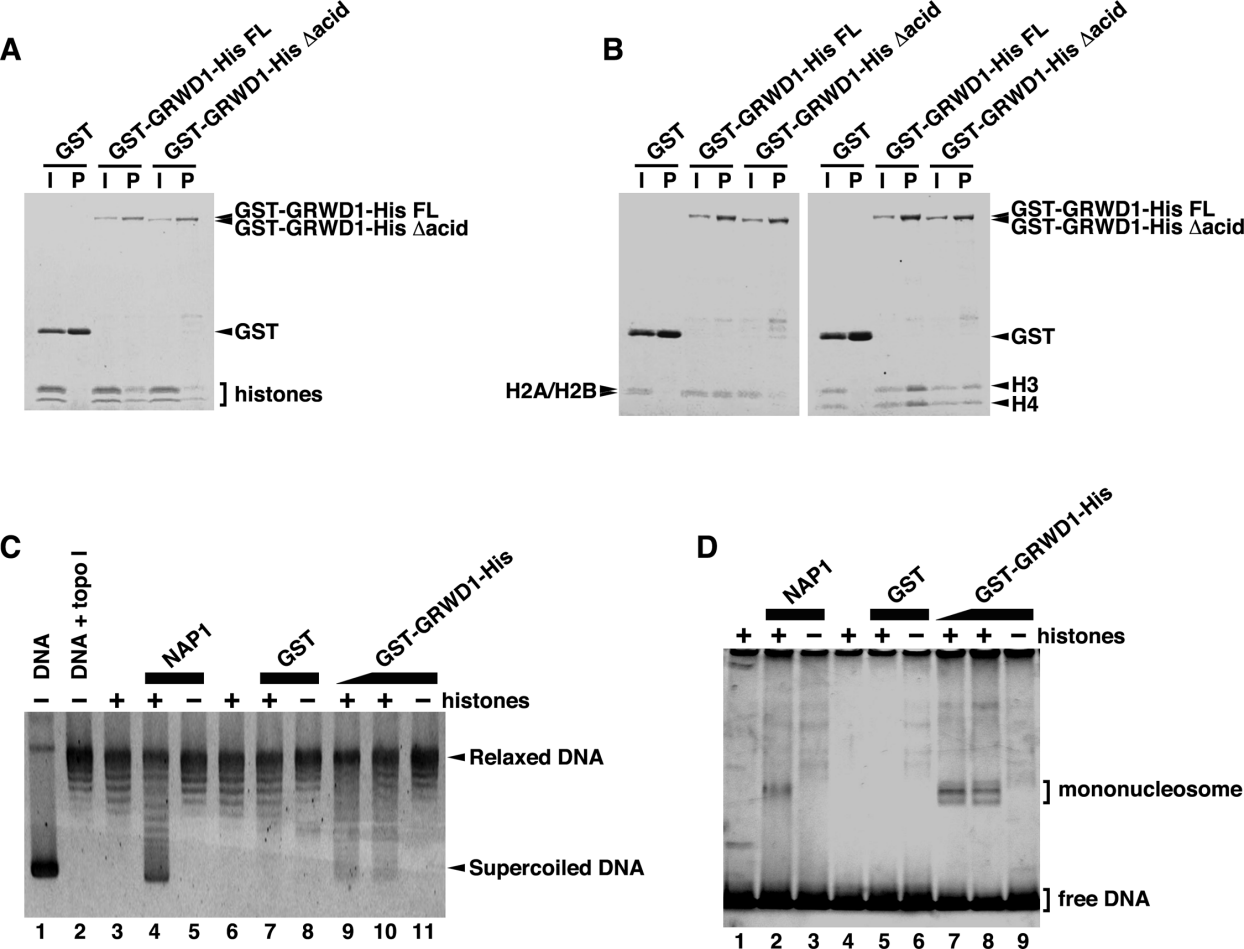
GRWD1 Has a Histone-Binding Activity. (**A**) GST-GRWD1-His, its truncated mutant GST-GRWD1-His Δacid, or GST was mixed with core histones purified from HeLa cells, and proteins bound to glutathione sepharose beads (P) with the input samples (I) were analyzed by CBB staining. (**B**) GST-GRWD1-His, GST-GRWD1-His Δacid, or GST was mixed with H2A/H2B or H3/H4 complexes, and bound proteins were subjected to CBB staining. (**C**) Topological assay was carried out with the indicated proteins. Supercoiled DNA (lane 1), DNA relaxed by topoisomerase I (lane 2), relaxed DNA incubated with reaction buffer alone, NAP1 (1 μM), GST (1 μM) or GST-GRWD1-His (0.5 μM or 1 μM), in the presence or absence of core histones, was analyzed on an agarose gel electrophoresis followed by SYBR Gold staining. (**D**) Mononucleosome assembly assay was performed with the indicated proteins. A 195 bp 5S DNA incubated with reaction buffer alone, NAP1 (1 μM), GST (1 μM) or GST-GRWD1-His (0.5 μM or 1 μM), in the presence or absence of recombinant histones, was analyzed by non-denaturing PAGE.

Given its two characteristic domains (Figure 1A), we speculated that GRWD1 might bind to histones. Actually, we found that histone octamers purified from HeLa cells bind to recombinant GST-GRWD1-His proteins (Figure 4A and Supplementary Figure S4A). We also prepared recombinant human H2A/H2B and H3-H4 complexes from *Escherichia coli* and repeated the assay. As shown in Figure 4B, GRWD1 interacted with both H2A/H2B and H3-H4. In addition, we identified histone proteins as GRWD1-binding proteins in a proteomics approach using GRWD1 immunoprecipitates (data to be published elsewhere). Histone binding was reduced by approximately half after deletion of the acidic domain (Figure 4A and B), but was not affected by deletion of the C-terminus (Supplementary Figure S4B).

Given its binding to histones, we hypothesized that GRWD1 might have histone-chaperone activity. We first performed topological assays (32,33) with purified GST-GRWD1-His (Supplementary Figure S4A). The formation of core particle in this assay is reflected by faster electrophoretic migration of initially relaxed plasmid DNA. The incubation of histones with GST-GRWD1-His led to histone deposition onto plasmid DNA (Figure 4C, lanes 9–10), which was not achieved by GST alone (Figure 4C, lane 7). NAP1 histone chaperone efficiently loaded histones onto DNA in this assays; the activity of GRWD1 appeared to be lower than that of NAP1. We also tested the nucleosome-assembly activity of GRWD1 in a nucleosome-formation assay, using a short DNA fragment (32,33). As shown in Figure 4D, mononucleosomes were formed in the presence of GST-GRWD1-His. Although further confirmation with the untagged proteins would be required, these results suggest that GRWD1 might have a histone chaperone-like activity.

### GRWD1 co-localizes with pre-RC genome wide

We wanted to know whether GRWD1 co-localizes with pre-RC genome wide. In metazoan cells, the pre-RC may not form strictly on specific sites due to the low sequence specificity of ORC DNA binding and the redundant and degenerated regulation in determination of pre-RC sites. It is now believed that the pre-RC may be formed with some preferentiality, albeit stochastically, in metazoan chromatin (34–38). Indeed, in our qPCR assay on the *lamin B2* and *MCM4* loci, the data indicate that the pre-RC may be formed preferentially on ‘origin’ regions, but also at lower frequency on the origin–distal regions. Overall, genome wide distribution of preferred pre-RC sites or pre-RC-enriched regions remains elusive in human cells. We addressed this end by performing ChIP-deep sequencing (Seq) with utilizing aggregation plots (25). For quality control of our ChIP-Seq analysis, see also Supplementary Figure S5A, B and D.

**Figure 5.**
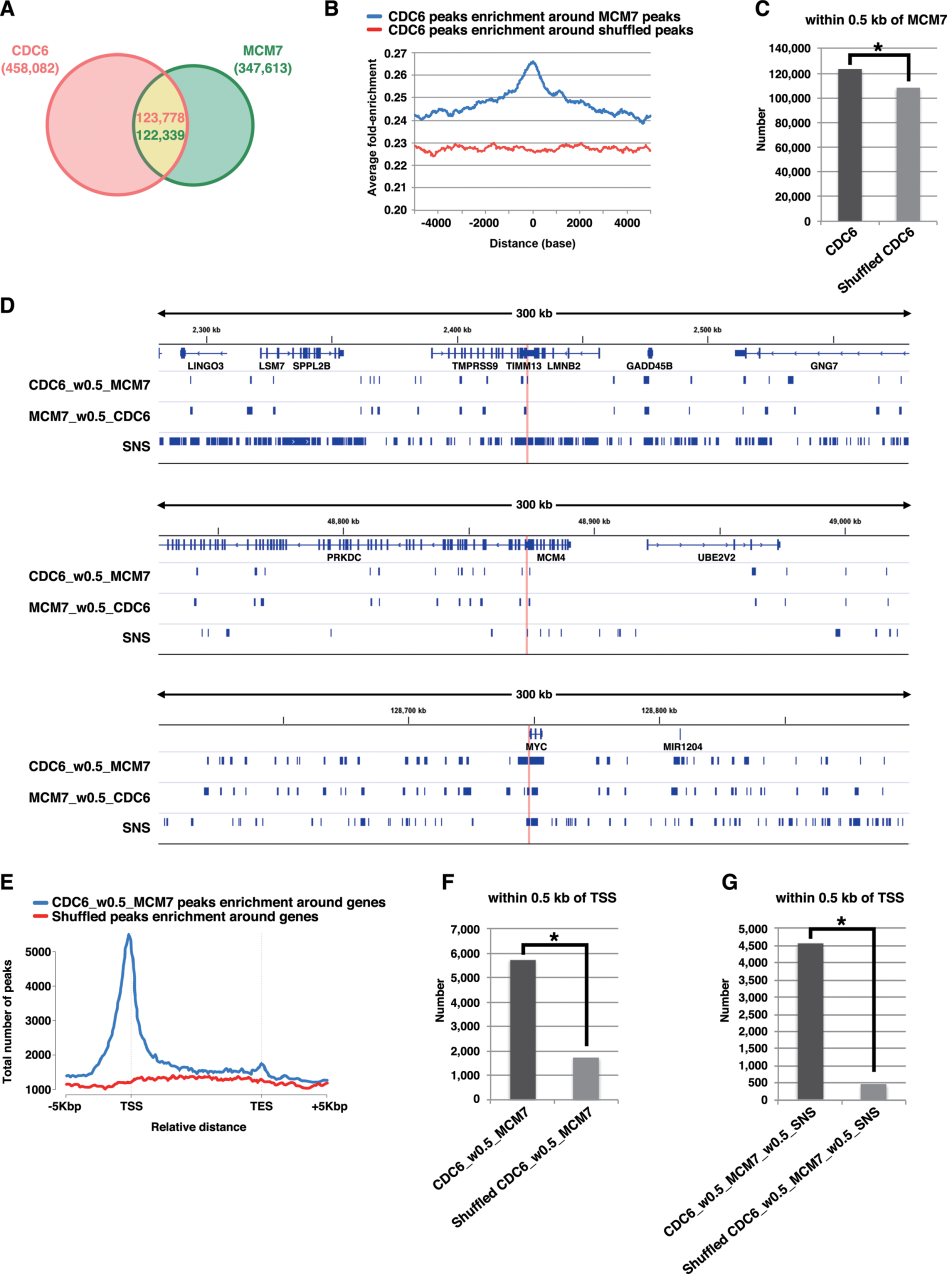
Genome wide identification of pre-RC-enriched zones. (**A**) Venn diagram showing the numbers of CDC6-binding or MCM7-binding peaks (called by MACS2 broad peak caller in ChIP-Seq for asynchronous HeLa cells) and their overlap within 0.5 kb. (**B**) CDC6 binding peaks were aggregated for MCM7 binding sites (peaks). The average fold-enrichment of CDC6 was plotted relative to the position of all MCM7 binding sites. The ‘shuffled MCM7 peaks’ were randomly chosen from genome. (**C**) The number of CDC6 peaks within 0.5 kb of MCM7 peaks is significantly higher than that obtained with randomized CDC6 peaks. *, *P* < 0.001 by Chi-Square test. (**D**) Distributions of the selected CDC6 (i.e. within 0.5 kb of MCM7) and MCM7 (i.e. within 0.5 kb of CDC6) peaks on genomic loci containing the *LMNB2, MCM4* or *c-myc* genes. Positions of ‘preferential origins’ are indicated. The data for SNS peaks of HeLa cells were derived from a paper by Besnard *et al*. (39). (**E**) The frequency of the pre-RC sites (the selected CDC6 peaks that are within 0.5 kb of MCM7 peaks) at all genes was calculated to assess the distribution of the pre-RC sites on genes. Marks -5 kbp and +5 kbp indicate 5 kb upstream of TSSs (Transcription start sites) and 5 kb downstream of TESs (Transcription end sites), respectively. (**F**) The number of the pre-RC sites (i.e. the selected CDC6 peaks within 0.5 kb of MCM7 peaks) within 0.5 kb of TSS are significantly higher than that obtained with the randomized pre-RC sites. *, *P* < 0.001 by Chi-Square test. (**G**) The number of the firing pre-RC sites (i.e. the selected CDC6 peaks which are within 0.5 kb of MCM7 peaks and SNS peaks) within 0.5 kb of TSS are further higher than that obtained with the randomized peaks. *P < 0.001 by Chi-Square test.

To determine preferred pre-RC sites accurately, we performed ChIP-Seq for both CDC6 and MCM7 using asynchronous HeLa cells (see also Supplementary Figure S6 for visual representation of the ChIP-Seq signal patterns for some representative genomic loci). MACS2 broad peak caller identified ∼458 000 peaks of CDC6 binding (*P* < 0.001, *q* < 0.05) and ∼347 000 peaks of MCM7 binding (*P* < 0.001, *q* < 0.005) (Figure 5A). For accuracy of our MCM7 mapping, we confirmed that the MCM7 peaks are closely associated with short nascent strand (SNS) peaks, which may represent firing replication origins (39) (Supplementary Figure S5B). We then analyzed the association of CDC6 peaks with MCM7 peaks. In analysis of CDC6 peak distribution relative to MCM7 peak, significant enrichment of the CDC6 peaks was found around MCM7 peaks with a central sharp peak (Figure 5B). This pattern was lost when using the randomized (shuffled) data sets, which contain the same number and length of the corresponding data sets but are randomly located, to estimate correlation by chance, indicating the significance. Even if a stringent overlap criterion of 0.5 kb was used to consider co-localization, approximately ∼27% (∼123 000 peaks) of total CDC6 peaks were accompanied by MCM7 peaks (Figure 5A). This association was statistically significant when compared with the data obtained with randomized CDC6 peaks (Figure 5C). Hereafter, we use the term ‘CDC6_w0.5_MCM7’ as representing CDC6 peaks that are accompanied by MCM7 peaks within 0.5 kb and ‘CDC6_wo0.5_MCM7’ as representing the remaining CDC6 peaks that are not accompanied by MCM7 peaks within 0.5 kb. In visual inspection for three genomic regions, *lamin B2, MCM4* and *c-myc* loci, the selected CDC6 peaks (CDC6_w0.5_MCM7) and MCM7 peaks (MCM7_w0.5_CDC6) appeared to be clustered on some specific regions, forming pre-RC-enriched domains including known origins (Figure 5D). Given that the size of the human genome is ∼3 × 10^9^ bp, it can be estimated that the selected CDC6 peaks were distributed with an average interval of ∼25 kb. Note that this does not simply mean that pre-RCs are formed once per ∼25 kb in a single cell. Rather, it would mean that the sites on which pre-RCs potentially can be formed are spaced ∼25 kb apart on average but in each cell, pre-RCs are actually formed on some of these sites stochastically with some preferentiality (34–38). In the ∼170 kb Epstein-Barr virus genome, 64 pre-RC-enriched zones can be detected with ChIP-microarray analysis (37). In *Drosophila* cells, median distance between ORC binding sites is estimated to be ∼11 kb (36). On the other hand, it is thought that only a limited number of pre-RCs are fired during a given S phase (37,40–41). In general, the number of the selected pre-RC peaks may be consistent with reasonable expectations. Recent genome-wide studies in human, mouse and fly cells reveal that origins often co-localize with TSSs (35–36,38). We also found that the selected CDC6 binding peaks are enriched in the genomic segments near the TSSs (Figure 5E and F). Furthermore, the firing pre-RC sites (namely, CDC6_w0.5_MCM7_w0.5_SNS; total ∼32 000 sites; see Supplementary Figure S5C) seem to be more closely associated with TSS (Figure 5G). Together, it is likely that most of the selected CDC6 and MCM7 peaks may represent bona fide pre-RC sites in HeLa cells.

**Figure 6.**
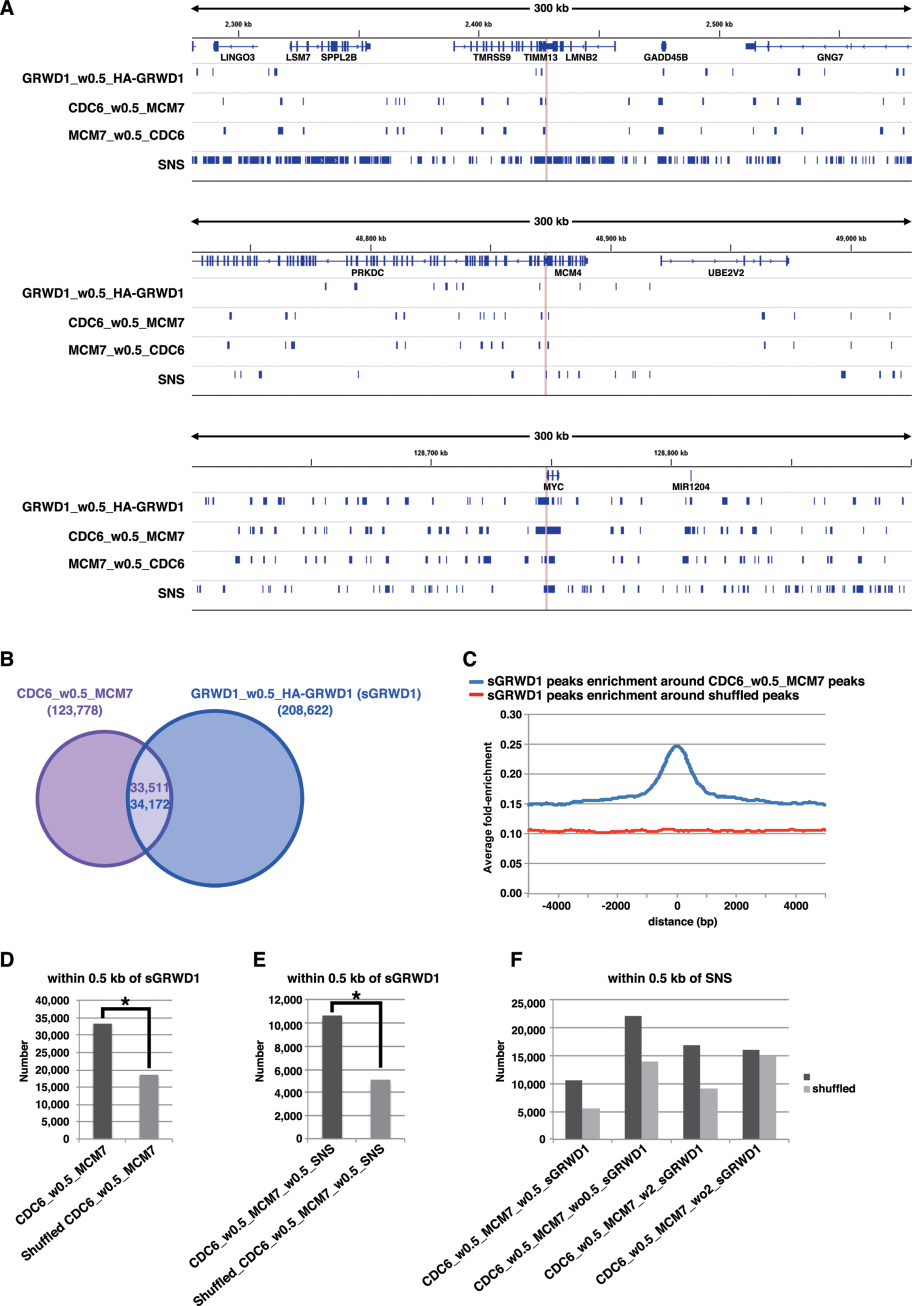
GRWD1 co-localizes with pre-RC genome wide. (**A**) Visual representation of the GRWD1-binding sites (the selected GRWD1 [sGRWD1] peaks, which are within 0.5 kb of HA-GRWD1 peaks) from asynchronous HeLa cells for genomic loci containing the *LMNB2, MCM4* or *c-myc* genes. For comparison, the data shown in Figure 5D are re-presented in parallel. (**B**) Venn diagram showing the numbers of the pre-RC sites (CDC6_w0.5_MCM7) or the selected GRWD1-binding sites and their overlap within 0.5 kb. (**C**) The selected GRWD1 binding sites (peaks) were aggregated for the pre-RC sites (CDC6_w0.5_MCM7). The average fold-enrichment of GRWD1 was plotted relative to the position of the pre-RC sites. (**D**) The number of the pre-RC sites within 0.5 kb of the selected GRWD1 peaks is significantly higher than that obtained with the randomized pre-RC sites. *, *P* < 0.001 by Chi-Square test. (**E**) The number of the firing pre-RC sites (CDC6 peaks that are within 0.5 kb of MCM7 and SNS) within 0.5 kb of the selected GRWD1 peaks is significantly higher than that obtained with the randomized firing pre-RC sites. *, *P* < 0.001 by Chi-Square test. (**F**) The pre-RC sites with GRWD1 (CDC6_w0.5_MCM7_w0.5_sGRWD1) appear to be more closely associated with SNS than the pre-RC sites without GRWD1.

Similarly, genome-wide GRWD1 localization in asynchronous HeLa cells was examined (Supplementary Figure S5D and S6). Consequently, ∼208 000 GRWD1 binding peaks were selected (Figure 6A and B). We then analyzed the association of the selected CDC6 peaks (CDC6_w0.5_MCM7) with the selected GRWD1 (sGRWD1) peaks (GRWD1_w0.5_HA-GRWD1). As a result, ∼27% (∼33 000) of the selected CDC6 peaks (pre-RC peaks) were found to be significantly co-localized at or near (i.e. within 0.5 kb of) the GRWD1 peaks (Figure 6A–D). Such regions include the *lamin B2, MCM4* and *c-myc* origins (Figure 6A). The firing pre-RC sites were also found to be significantly associated with GRWD1 peaks (Figure 6E). These data indicate that GRWD1 co-localizes with the pre-RC, not only at specific origins but also genome wide. The pre-RC sites with GRWD1 (CDC6_w0.5_MCM7_w0.5_sGRWD1) appear to be more closely associated with SNS (Figure 6F). Since the firing pre-RC sites with GRWD1 (CDC6_w0.5_MCM7_w0.5_sGRWD1_w0.5_SNS) have open chromatin structure (see below), it could be that such open chromatin structure facilitates origin firing by enhancing recruitment of initiation proteins such as CDC45. On the other hand, these data also indicate that GRWD1 is not recruited to all pre-RC sites. In addition, the ChIP-Seq data also indicate that GRWD1 is distributed throughout the genome independent of pre-RC, where it could play other roles, e.g. in transcriptional control.

### GRWD1 controls chromatin structure at pre-RC sites

It seemed likely that GRWD1 regulates chromatin dynamics as a histone-binding protein. Therefore, we finally characterized the chromatin architecture around pre-RC sites and understand the effect of GRWD1 depletion. We here used FAIRE (formaldehyde-assisted isolation of regulatory elements), a method of isolating genomic regions with depleted or coarse nucleosomes (42–44).

**Figure 7.**
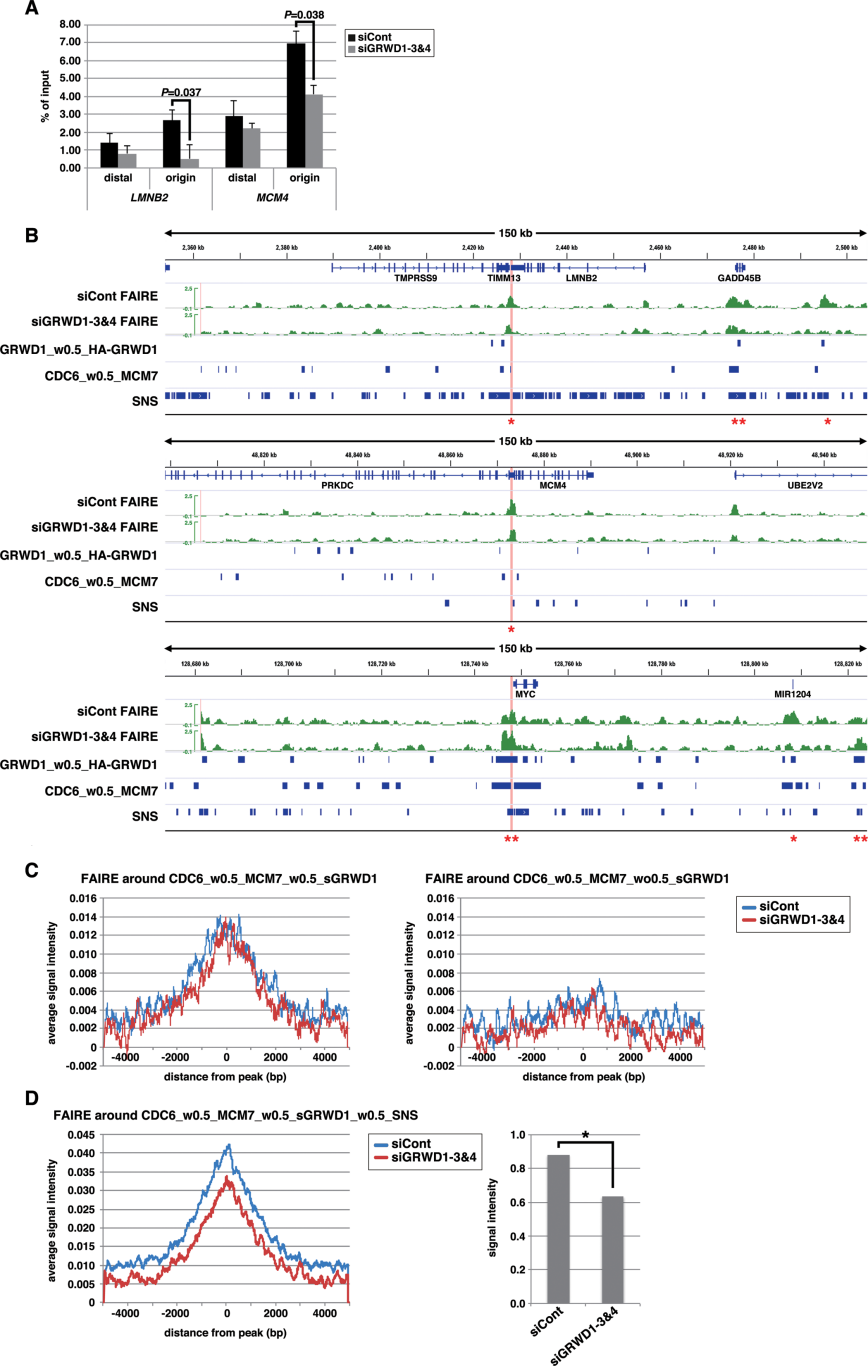
GRWD1 affects chromatin architecture (chromatin openness) at pre-RC sites. (**A**) GRWD1 depletion reduces chromatin openness at the replication origins at the *lamin B2* and *MCM4* loci. Asynchronous HeLa cells were treated with control or GRWD1 (mixture of siGRWD1–3&4) siRNAs for 48 h and subjected to FAIRE-qPCR. The means ± SD are shown (*n* = 3). (**B**) Visual representation of FAIRE-Seq results for genomic loci containing the *LMNB2, MCM4* or *c-myc* genes. The FAIRE DNA from HeLa cells treated with control (mixture of siLuci&siGFP) or GRWD1 (mixture of siGRWD1–3&4) siRNAs for 48 h was subjected to Seq. For comparison, the data shown in Figures 5D and 6A are re-presented in parallel. *Examples of the pre-RC sites with GRWD1 where FAIRE profiles appear to be affected by GRWD1 depletion. **Examples of the pre-RC sites with GRWD1 where FAIRE profiles appear not to be affected by GRWD1 depletion. (**C**) Cumulative FAIRE signal profiles at the pre-RC sites with GRWD1 (CDC6_w0.5_MCM7_w0.5_sGRWD1) (left) and those without GRWD1 (right) are shown for either control or GRWD1 siRNA-treated HeLa cells. For both sites, 10 000 sites were randomly selected from the corresponding data sets and used to adjust the peak numbers for the aggregation plots. (**D**) GRWD1 depletion significantly affects FAIRE profiles around the firing pre-RC sites with GRWD1. Cumulative FAIRE signal profiles at the firing pre-RC sites with GRWD1 (CDC6_w0.5_MCM7_w0.5_sGRWD1_w0.5_SNS; 10 631 peaks) are shown for either control or GRWD1 siRNA-treated HeLa cells (left). Average FAIRE signal of the 10 631 peaks (± 2 kb from the center of each peaks) from GRWD1-depleted cells is significantly lower than that from control siRNA-treated cells (right panel). *, *P* < 0.001.

We first confirmed the quality of our FAIRE analysis in several criteria (Supplementary Figure S7A–C). We then investigated quantitative FAIRE signals by qPCR on *lamin B2* and *MCM4* origins, revealing that the signals at these regions were decreased upon GRWD1 depletion (Figure 7A). Similar results were observed with another siRNA targeting GRWD1 (Supplementary Figure S7D).

We next analyzed the genome-wide FAIRE signal profiles and compared it with that from the GRWD1-depleted cells. In a visual inspection of the data, the origin regions of *lamin B2, MCM4* and *c-myc* loci showed prominent positive signals, indicating that these origins are in open chromatin structure (Figure 7B). In addition, some candidate firing origins appear to be in open chromatin structure, as well (Figure 7B). The FAIRE-Seq profiles for some pre-RC sites with GRWD1, including *lamin B2* and *MCM4* origins, appear to be altered by GRWD1 depletion, whereas the others, including *c-myc* origin, appear not to be affected by GRWD1 depletion (Figure 7B). We examined the aggregated FAIRE signals around the identified pre-RC sites with or without GRWD1 to know the average statuses. The data clearly indicate that the pre-RC sites with GRWD1 (CDC6_w0.5_MCM7_w0.5_sGRWD1) have more open chromatin structure than the latter (Figure 7C). However, GRWD1 depletion did not obviously change the aggregated FAIRE profile at the pre-RC sites with GRWD1 (Figure 7C). As suggested above at some representative origins (Figure 7B), the firing pre-RC sites with GRWD1 might have further open chromatin structure and might be affected more strongly by GRWD1 depletion. We therefore examined the aggregated FAIRE signals around the CDC6_w0.5_MCM7_w0.5_sGRWD1_w0.5_SNS. The data suggest that the openness of chromatin at the firing pre-RC sites with GRWD1 is further increased, which is suppressed by GRWD1 depletion (Figure 7D). On average, ∼30% reduction in the FAIRE signals was observed for the firing pre-RC sites with GRWD1 (Figure 7D). Taken together, these data suggest that GRWD1 contributes to maintenance of chromatin openness genome wide. To gain further support for the notion obtained with our ChIP-Seq and FAIRE-Seq analyses, we performed following experiments. Referring to the Seq data, we picked two new candidates of replication origins regulated by GRWD1; i.e. two CDC6_w0.5_MCM7_w0.5_GRWD1_w0.5_SNS sites which may have GRWD1-dependent open chromatin structure. Here, these sites are tentatively termed *PAF1* origin and *LRIG2* origin, respectively (Supplementary Figure S8A). We examined quantitative ChIP signals and FAIRE signals by qPCR at these sites. Like *LMNB2* and *MCM4* origins, the origin sequences were significantly enriched with anti-CDC6, anti-Cdt1, anti-MCM7, anti-MCM3 and anti-GRWD1 antibodies (Supplementary Figure S8B and C). As is the case for *LMNB2* and *MCM4* origins, some enrichment of the origin-distal regions was also observed, but less efficient. Furthermore, silencing of GRWD1 decreased the GRWD1 and MCM ChIP signals, but not CDC6 and Cdt1 signals (Supplementary Figure S8B and C). These data indicate that pre-RCs are formed on these origins in a GRWD1-stimulated manner. We then carried out FAIRE-qPCR assay (Supplementary Figure S8D and E). The data indicate that the origin sites have open chromatin structure and GRWD1 plays a role in maintaining the chromatin openness, like *LMNB2* and *MCM4* origins.

## DISCUSSION

Here, we show that GRWD1 is recruited to the *lamin B2* and *MCM4* locus origins, in a CDC6- and Cdt1-dependent manner (Figure 2 and Supplementary Figure S2), and stimulates MCM loading (Figure 3 and Supplementary Figures S3 and S8). Throughout the genome, GRWD1 may co-localize to ∼27% of pre-RC sites (Figure 6). In accordance with this, GRWD1 depletion has an influence on replication and genome integrity (Figure 3 and Supplementary Figure S3). At this time, it remains unclear how GRWD1 recruitment to specific pre-RCs is dictated. Some chromatin architectures and/or existence of additional factor(s) would affect the selection. Upon pre-RC sites not associated with GRWD1, other proteins might function as cofactors to stimulate MCM loading.

We found that GRWD1 has histone-binding and histone chaperone-like activities (Figure 4 and Supplementary Figure S4). Given the activity and recruitment to certain pre-RCs, it is conceivable that GRWD1 may enhance chromatin accessibility at these sites. Our FAIRE analyses suggest that at least on some, but not all, GRWD1-bound pre-RC sites, including the *lamin B2* and *MCM4* origins, this may be indeed the case (Figure 7 and Supplementary Figures S7 and S8). At unaffected GRWD1-bound pre-RC sites, other proteins might redundantly function. At the *lamin B2* and *MCM4* origins, changes in total histone H3 levels were not detected upon GRWD1 depletion (Supplementary Figure S7E). Therefore, it is possible that GRWD1, in cooperation with HBO1 and SNF2H, might relax histone-DNA interactions to increase the ratio of nucleosome-free DNA rather than simply evict most histones. Similar findings were observed at rDNA origins in fission yeast, where depletion of Sir2 HDAC may result in open chromatin structure, apparently without changing histone levels and positioning (45).

Many co-factors that stimulate pre-RC formation have been reported. Several factors, such as the Myb, c-myc and E2F transcription factors, as well as ORCA, enhance chromatin loading of ORC and/or CDC6 (46–49). These factors may stabilize DNA binding of the MCM loaders. Another class of co-factors includes those that are recruited to origins by interaction with ORC, CDC6 and/or Cdt1 to enhance MCM loading. For example, the HBO1 and SNF2H are recruited to origins in a Cdt1-dependent manner (13–15). The execution point of GRWD1 is similar to these two factors, and all of them are recruited to the pre-RC via Cdt1. Therefore, we now favor a model in which these factors function cooperatively. However, it is also possible that each factor acts individually on specific origins. In addition, at this time, we cannot completely exclude the possibility that GRWD1 stimulates MCM loading through other unidentified pathways.

Our ChIP-Seq analysis gives some insight into the genome-wide regulation of pre-RC sites in human cells. Metazoan ORC does not bind to DNA in a sequence-specific manner; therefore, pre-RC binding sites may be determined by the chromatin environment in a redundant and degenerate fashion, as suggested by recent genome-wide studies (36,37). Pre-RCs tend to be formed at open chromatin regions without common sequence motifs. Overall, our data for pre-RC sites (Figure 5) appear to be in line with this notion. Although open chromatin may promote ORC binding, it may not be sufficient, and additional proteins as above and/or specific DNA structures, such as negative supercoiling may also be required. However, it seems unlikely that only a limited number of factors execute functions in ORC and/or pre-RC recruitment. It is possible that suitable factors are selected for pre-RC recruitment in a manner that depends on the local chromatin environment.

Given that GRWD1 is a histone-binding protein regulating chromatin throughout the genome, it may function also in other DNA transactions. In support of this notion, we have identified several transcription factors and chromatin factors as novel GRWD1 binding proteins (data to be published elsewhere). In addition, GRWD1 is required for maintenance of genome integrity, because its depletion leads to DNA damage (Figure 3). Furthermore, we have observed GRWD1 overexpression in cancer cells (Supplementary Figure S1C), also suggesting its importance in promotion of cell growth. Taken together, our findings warrant future in-depth study of GRWD1.

## ACCESSION NUMBERS

ChIP-seq and FAIRE-Seq data were deposited with accession codes DRA000924, DRA001271 and DRA002539.

### SUPPLEMENTARY DATA

Supplementary Data are available at NAR Online.

## Supplementary Material

SUPPLEMENTARY DATA
