# Supplementary material for: Cdt1-binding protein GRWD1 is a novel histone-binding protein that facilitates MCM loading through its influence on chromatin architecture
Source: Nucleic Acids Res. 2015 May 18;43(12):5898–911. doi: 10.1093/nar/gkv509 (PMC4499137; doi:10.1093/nar/gkv509)
Supplement: SUPPLEMENTARY DATA [file supp_43_12_5898__index.html]

Cdt1-binding protein GRWD1 is a novel histone-binding protein that facilitates MCM loading through its influence on chromatin architecture — SUPPLEMENTARY DATA 

# Cdt1-binding protein GRWD1 is a novel histone-binding protein that facilitates MCM loading through its influence on chromatin architecture

## SUPPLEMENTARY DATA

- SUPPLEMENTARY DATA
