# Supplementary material for: Cdt1-binding protein GRWD1 is a novel histone-binding protein that facilitates MCM loading through its influence on chromatin architecture
Source: Nucleic Acids Res. 2015 May 18;43(12):5898–911. doi: 10.1093/nar/gkv509 (PMC4499137; doi:10.1093/nar/gkv509)
Supplement: SUPPLEMENTARY DATA [file supp_gkv509_GRWD1_Supplement_NAR_ver3.2.pdf]

SUPPLEMENTARY INFORMATION

Cdt1-Binding Protein GRWD1 Is a Novel Histone-Binding Protein that Facilitates MCM Loading through its influence on chromatin architecture

Nozomi Sugimoto, Kazumitsu Maehara, Kazumasa Yoshida, Shuhei Yasukouchi, Satoko Osano, Shinya Watanabe, Masahiro Aizawa, Takashi Yugawa, Tohru Kiyono, Hitoshi Kurumizaka, Yasuyuki Ohkawa, and Masatoshi Fujita

Supplementary information includes 8 Figures, Supplementary Materials and Methods and Supplementary References.

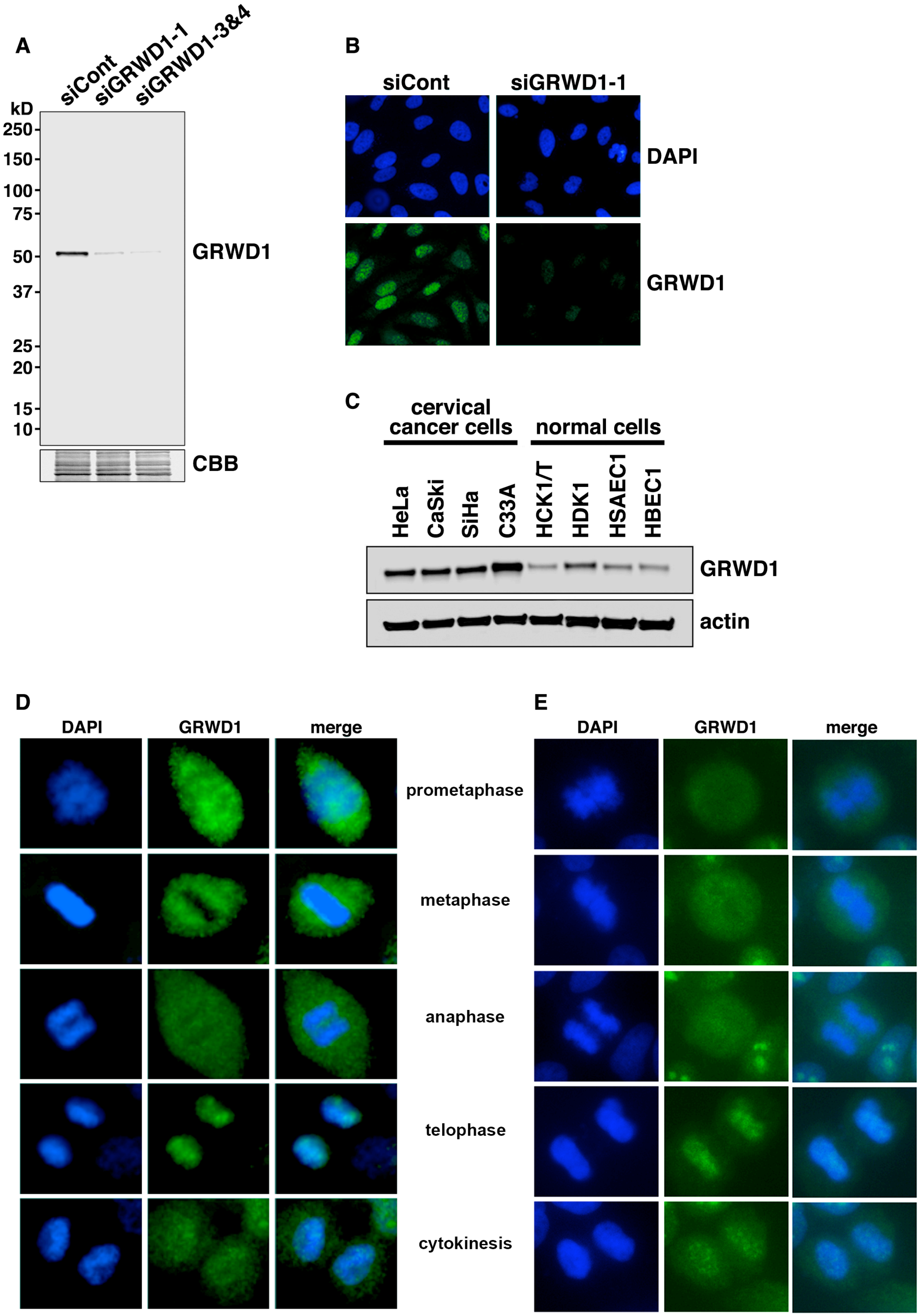
A
siCont
siGRWD1-1
siGRWD1-3&4
kD
250
150
100
75
50
37
25
20
15
10
GRWD1
CBB
B
siCont
siGRWD1-1
DAPI
GRWD1
C
cervical cancer cells
normal cells
HeLa
CaSki
SiHa
C33A
HCK1/T
HDK1
HSAEC1
HBEC1
GRWD1
actin
D
DAPI
GRWD1
merge
prometaphase
metaphase
anaphase
telophase
cytokinesis
E
DAPI
GRWD1
merge

Supplementary Figure S1. Detection of GRWD1 Proteins in Immunoblotting and Immunostaining Using Anti-GRWD1 Antibody

(A) Whole-cell lysates from HeLa cells transfected with control or GRWD1 (siGRWD1-1 or mixture of siGRWD1-3&4) siRNAs for 48 hr were immunoblotted with anti-GRWD1 antibodies.

(B) HeLa cells transfected with siRNAs as in (A) were immunostained with anti-GRWD1 antibodies, counterstained with DAPI, and analyzed.

(C) GRWD1 Overexpresses in Cancer Cell Lines. Several DNA replication-initiation proteins are overexpressed in cancer cell lines (29, 30, 31). GRWD1 has been implicated in ribosomal biogenesis, and here we show its involvement in DNA replication control and possibly in other chromatin transactions. GRWD1 was also identified as a candidate substrate-receptor of Cul4-DDB1 ubiquitin ligase. Taken together, these observations suggest that GRWD1 plays important roles in control of cell growth. Therefore, we compared the expression levels of GRWD1 proteins in human cancer cells and normal human cells. Whole-cell extracts were prepared from several cervical-cancer cell lines (HeLa, CaSki, SiHa, and C33A) and normal epithelial cell lines (HCK1/T, HDK1, HSAEC1, and HBEC1) (30), and immunoblotted with the indicated antibodies. Actin was used as a loading control. On average, GRWD1 protein levels in the cancer cells were 5-20 times greater than those in normal cells. The findings seem consistent with the notion that GRWD1 is involved in regulation of cell growth.

(D, E) Localization of GRWD1 proteins during M phase. (D) HeLa Cells were fixed with 3.7% formaldehyde and probed with anti-GRWD1 antibody. The samples were then incubated with Alexa Fluor 488-conjugated goat anti-rabbit IgG antibody, counterstained with DAPI, and analyzed. (E) Similar immunostaining analysis was performed after pre-extraction with 0.1% Triton X-100 in PBS.

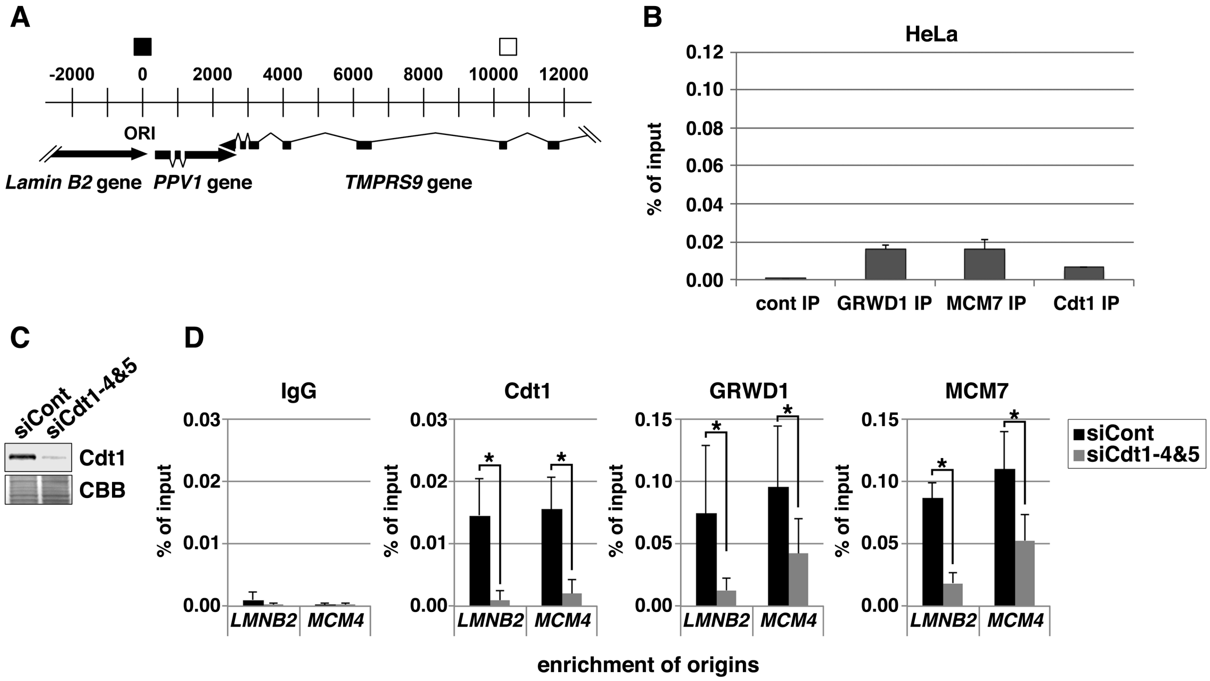

Supplementary Figure S2. Additional Data for Cdt1-dependent Binding of GRWD1 to Replication Origins

(A) Pre-RC and GRWD1 proteins do not bind to a genomic region 10 kb apart from the lamin B2 origin. Referring to our own ChIP-Seq data (see below), we designed qPCR primers (5'-GACAGTGGGGAGACTCTTGC -3' and 5'-GACGAAGAAACCAGGGCTCA-3') to detect genomic DNA sequence (white box) that is ~10 kb apart from lamin B2 origin (black box) and is expected to be negative for pre-RC and GRWD1 bindings. (B) ChIP-qPCR assay for the indicated immunoprecipitates from asynchronously growing HeLa cells was performed with the above primers. Results are shown as the percent of input DNA. The means±S.D. are shown (n=3). (C, D) Silencing of Cdt1 with another siRNA (siCdt1-4&5) also inhibits recruitment of GRWD1 and MCM7 onto replication origins at the lamin B2 and MCM4 loci in HeLa cells. Cells transfected with control or Cdt1 (siCdt1-4&5) siRNAs for 48 hrs were subjected to immunoblotting (C) and ChIP-qPCR (D) with the indicated antibodies as described in Figure 2. For ChIP data, the means±S.D. are shown (n=4). *, $p<0.05$.

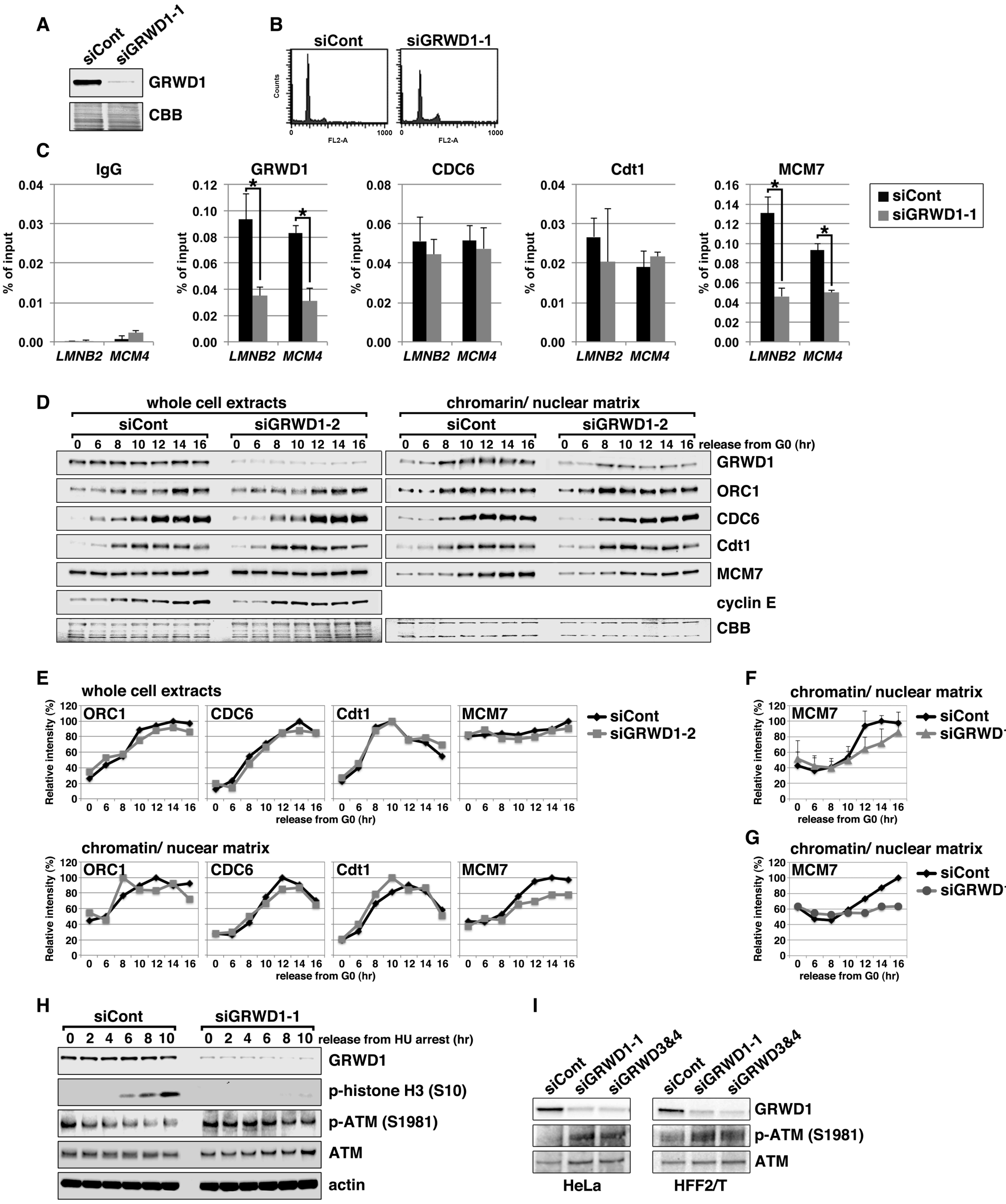

Supplementary Figure S3. Silencing of GRWD1 Reduces MCM Loading

(A-C) Silencing of GRWD1 with another siRNA (siGRWD1-1) also inhibits recruitment of MCM onto Lamin B2 or MCM4 locus origins in HeLa cells. Cells transfected with control or GRWD1 (siGRWD1-1) siRNAs for 48 hrs were subjected to immunoblotting (A), FACS (B),

and ChIP-qPCR (C) with the indicated antibodies as described in Figure 3. For ChIP data, the means±S.D. are shown (n=3). *, $p<0.05$.

(D-G) Silencing of GRWD1 partially suppresses MCM loading assessed by chromatin-binding assay in synchronized T98G cells. (D, E) Cells were transfected with control or GRWD1 (siGRWD1-2) siRNAs for 48 hrs, rendered quiescent by serum starvation for 48 hrs, and then stimulated to re-enter the cell cycle by serum addition (15). Cells were harvested at the indicated times and subjected to the chromatin-binding assay. (D) Whole-cell extracts and chromatin/matrix-binding fractions were immunoblotted. (E) The signal intensities of the pre-RC proteins were quantified, normalized to the signals of CBB staining, and shown with the maximum values set at 100 percent. (F, G) Similar experiments were performed with other siRNAs (siGRWD1-1 or siGRWD1-3) and the quantified data for MCM7 chromatin-bindings are shown. (F) Data obtained with siGRWD1-1. The means and standard deviations are shown (n=2). (G) Data obtained with siGRWD1-3. In control siRNA-treated cells, Cdt1 as well as CDC6, were absent after serum starvation. Levels of both proteins increased approximately 10 hrs after serum addition, coincident with the loading of MCM7 onto chromatin. For GRWD1, the total levels were not changed during the cell cycle; however, GRWD1 was detached from chromatin in G0 cells and rebounded when cells re-entered the cell cycle, suggesting its involvement in control of cell growth. Depletion of GRWD1 modestly but significantly reduced the recruitment of MCM7 onto chromatin, whereas CDC6 and Cdt1 loading were not affected. GRWD1 depletion did not affect the expression levels of ORC1, CDC6, Cdt1, MCM7, or cyclin E proteins under these experimental conditions.

(H) HeLa cells transfected with the indicated siRNAs for 24 hr were arrested in S phase with HU and released. The cells were then harvested at the indicated time points and analyzed by immunoblotting with indicated antibodies, as in Figure 3E.

(I) GRWD1 depletion induces ATM activation in asynchronously growing cells. HeLa or HFF2/T cells were transfected with the indicated siRNAs for 48 hr and then subjected to immunoblotting with the indicated antibodies.

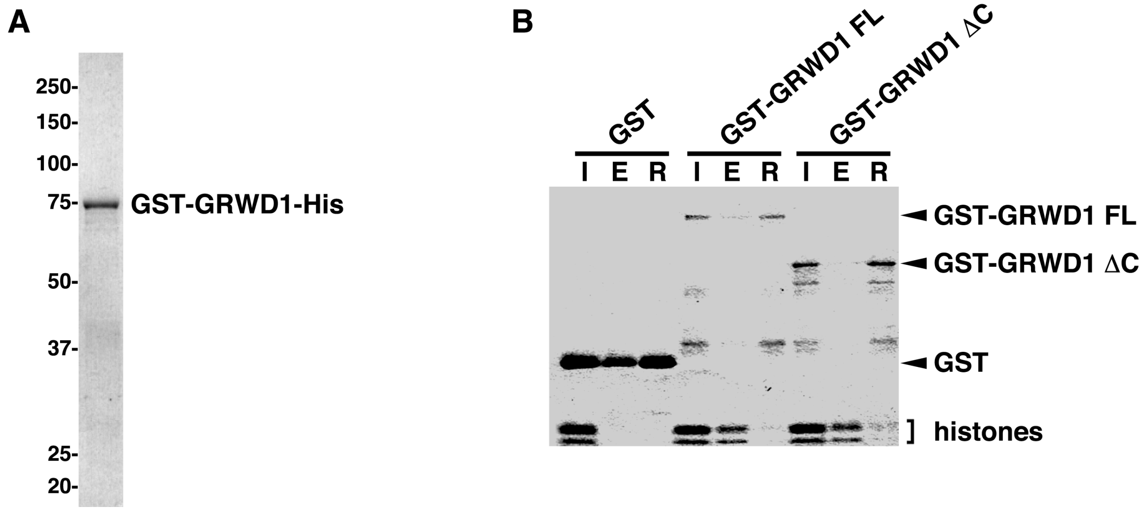

Supplementary Figure S4. The WD40 Repeat Domain Is Not Required for GRWD1 Binding to Histones

(A) Purification of bacterially produced GST-GRWD1-His. Bacterially produced GST-GRWD1-His proteins were purified as described in Experimental Procedures and analyzed on SDS-PAGE followed by CBB staining to confirm the purity.

(B) Full-length GST-GRWD1 (FL) or its truncated mutant GST-GRWD1 ΔC (lacking C-terminal amino acids 207-446) were incubated with the core histones, and bound proteins were first eluted with 1M NaCl (E: eluate) and then with SDS (R: remnant). The input sample (I) was also analyzed.

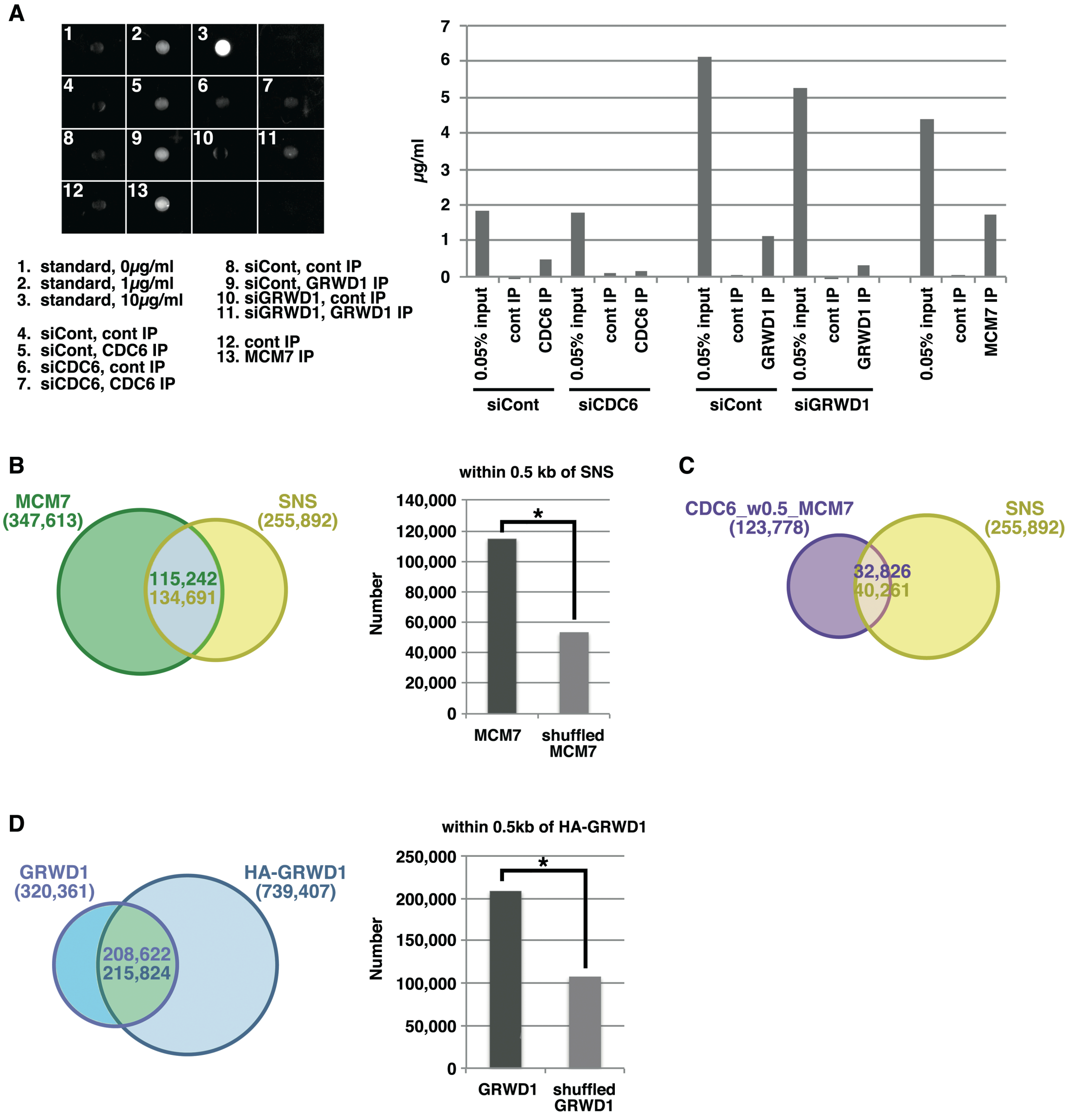

Supplementary Figure S5. Quality Control and Characterization of ChIP-Seq Analysis

(A) In our ChIP assays, most of the co-precipitated DNAs are specifically derived from the target proteins. We confirmed that remarkably higher amounts of chromatin DNA were precipitated with anti-CDC6, anti-MCM7, and anti-GRWD1 antibodies than control IgG and that the amounts of the co-precipitated DNA were significantly reduced upon treatment with GRWD1 or CDC6 siRNAs. 1-3, standard DNAs. 4-7, Co-precipitated DNAs were prepared from HeLa cells

transfected with control or CDC6 (siCDC6-1&3) siRNAs for 48 hr with anti-CDC6 antibody or control rabbit IgG. 8-11, Co-precipitated DNAs were prepared from HeLa cells transfected with control or GRWD1 (siGRWD1-3&4) siRNAs for 48 hr with anti-GRWD1 antibody or control rabbit IgG. 12-13, Co-precipitated DNAs were prepared from HeLa cells with anti-MCM7 antibody or control rabbit IgG. The DNAs were analyzed by SYBR Gold staining and UV photograph. The amounts of the co-precipitated DNAs were semi-quantified with the standard DNAs and are shown with the levels of input DNAs quantified by UV spectrometer (right panel).

(B) The MCM7 peaks we identified (~347,000 peaks; $p<0.001$, $q<0.005$) are closely associated with previously identified short nascent strand (SNS) peaks. To evaluate the accuracy of our pre-RC mapping, we utilized data sets from Seq analysis of SNS in HeLa cells (~255,000 peaks), which may represent firing replication origins (39). Since MCM complexes are believed to be replicative helicases essential for firing, we analyzed the association of MCM7 peaks with SNS peaks. Even if we used a stringent overlap criterion of 0.5 kb to consider co-localization, approximately ~52% (~134,000 peaks) of total SNS peaks were identified as those closely associated with the MCM7 peaks, and ~33% (~115,000) of MCM7 peaks as those closely associated with SNS (left panel). The value of MCM7 overlapping with SNS (~115,000) is ~2.1-fold higher than that obtained with the randomized (shuffled) data sets, which contain the same number and length of the corresponding data sets but are randomly located, to estimate correlation by chance (right panel). *, $p<0.001$ by Chi-Square test. Remaining MCM7 peak not associated SNS peaks may include dormant origins that do not fire or fire at low frequency.

(C) Identification of the firing pre-RC sites (CDC_w0.5_MCM7_w0.5_SNS). With a stringent overlap criterion of 0.5 kb to consider co-localization, ~32,000 peaks were identified as strong candidates for the firing pre-RC sites.

(D) Identification of GRWD1 binding peaks by ChIP-Seq. Genome-wide GRWD1 localization in asynchronous HeLa cells was examined by combining the analysis for endogenous GRWD1 using anti-GRWD1 antibody (see also Supplementary Figure S6) with that for exogenous HA-GRWD1 using anti-HA antibody. MACS2 broad peak caller identified ~320,000 GRWD1 peaks ($p<0.001$, $q<0.05$) and ~739,000 HA-GRWD1 peaks ($p<0.001$, $q<0.05$). We then analyzed the association of GRWD1 peaks with HA-GRWD1 peaks. Even when a stringent overlap criterion of 0.5 kb was used to consider co-localization, approximately ~65% (~208,000 peaks) of total GRWD1 peaks were identified as those closely associated with HA-GRWD1 peaks (left panel), the value being ~1.9-fold higher than that obtained with the shuffled data sets (right panel). *, $p<0.001$ by Chi-Square test. Hereafter, we used the selected GRWD1 (sGRWD1) peaks (namely, GRWD1_w0.5_HA-GRWD1) for further analyses.

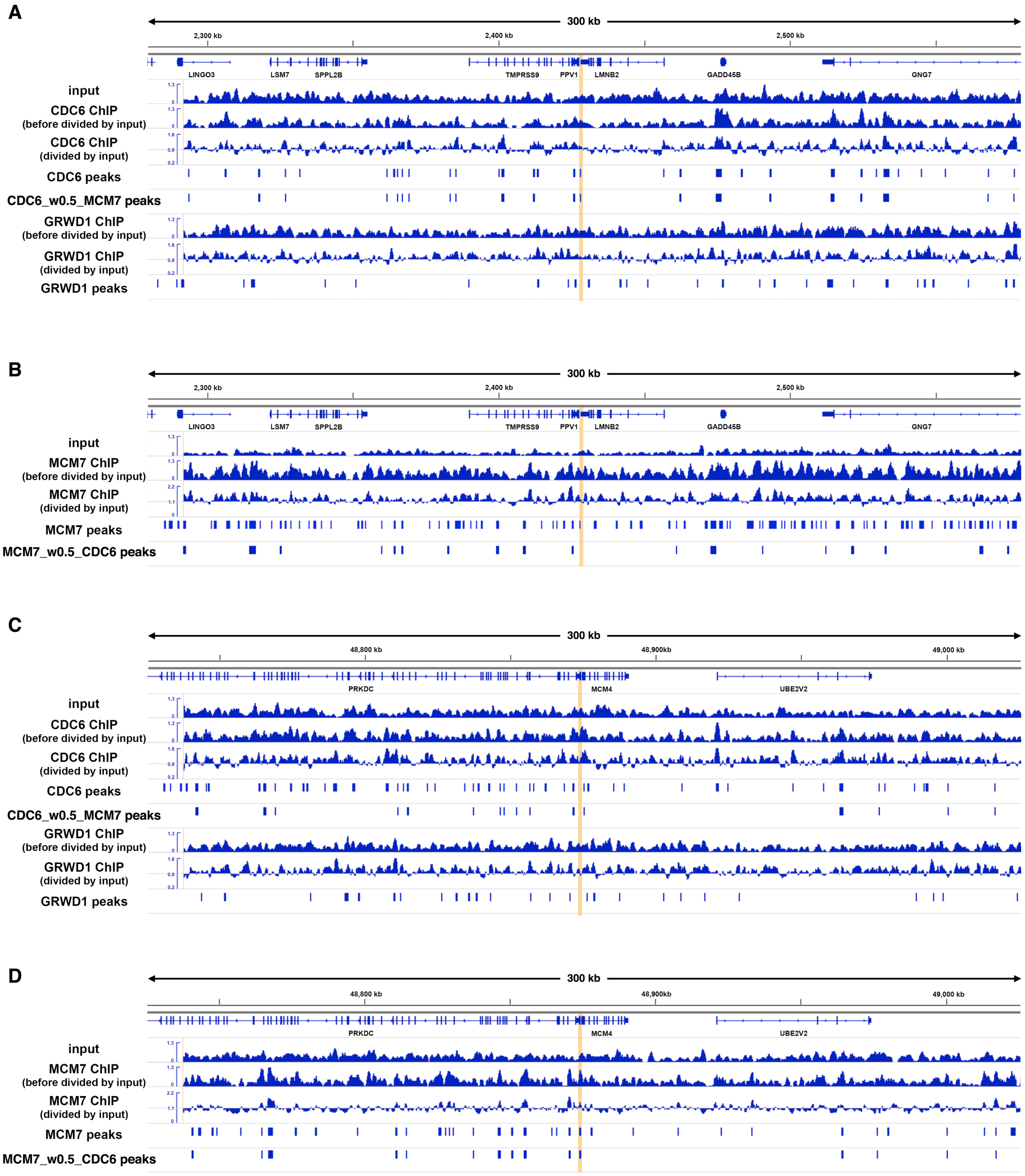
A
300 kb
2,300 kb
2,400 kb
2,500 kb
LINGO3
LSM7
SPPL2B
TMPRSS9
PPV1
LMNB2
GADD45B
GNG7
input
CDC6 ChIP
(before divided by input)
CDC6 ChIP
(divided by input)
CDC6 peaks
CDC6_w0.5_MCM7 peaks
GRWD1 ChIP
(before divided by input)
GRWD1 ChIP
(divided by input)
GRWD1 peaks
B
300 kb
2,300 kb
2,400 kb
2,500 kb
LINGO3
LSM7
SPPL2B
TMPRSS9
PPV1
LMNB2
GADD45B
GNG7
input
MCM7 ChIP
(before divided by input)
MCM7 ChIP
(divided by input)
MCM7 peaks
MCM7_w0.5_CDC6 peaks
C
300 kb
48,800 kb
48,900kb
49,000 kb
PRKDC
MCM4
UBE2V2
input
CDC6 ChIP
(before divided by input)
CDC6 ChIP
(divided by input)
CDC6 peaks
CDC6_w0.5_MCM7 peaks
GRWD1 ChIP
(before divided by input)
GRWD1 ChIP
(divided by input)
GRWD1 peaks
D
300 kb
48,800 kb
48,900kb
49,000 kb
PRKDC
MCM4
UBE2V2
input
MCM7 ChIP
(before divided by input)
MCM7 ChIP
(divided by input)
MCM7 peaks
MCM7_w0.5_CDC6 peaks

Supplementary Figure S6. Visual Representation of ChIP-Seq Results from Asynchronous HeLa Cells for Genomic Loci Containing the *LMNB2* or *MCM4* Genes.

(A) Visual representation of ChIP-Seq results for CDC6 and GRWD1 on *LMNB2* locus. Following data are shown. Seq data obtained with Input DNA sample (upper panel), primary Seq data obtained with CDC6 ChIP DNA sample (2nd panel), the data obtained by dividing the CDC6 ChIP-Seq data by the Input data (3rd panel), the MACS2 broad peak call annotation for CDC6 ChIP-Seq (4th panel), selected CDC6 peaks which are within 0.5 kb of MCM7 peaks (5th panel which is the same as shown in Figure 5D), primary Seq data obtained with GRWD1 ChIP DNA sample (6th panel), the data obtained by dividing the GRWD1 ChIP-Seq data by the Input data (7th panel), and the MACS2 broad peak call annotation for GRWD1 ChIP-Seq (8th panel).

(B) Visual representation of ChIP-Seq results for MCM7 on *LMNB2* locus. Following data are shown. Seq data obtained with Input DNA sample (upper panel), primary Seq data obtained with MCM7 ChIP DNA sample (2nd panel), the data obtained by dividing the MCM7 ChIP-Seq data by the Input data (3rd panel), the MACS2 broad peak call annotation for MCM7 ChIP-Seq (4th panel), and selected MCM7 peaks which are within 0.5 kb of CDC6 peaks (5th panel which is the same as shown in Figure 5D).

(C) Visual representation of ChIP-Seq results for CDC6 and GRWD1 on *MCM4* locus. The order of the data representation is the same as (A).

(D) Visual representation of ChIP-Seq results for MCM7 on *MCM4* locus. The order of the data representation is the same as (B).

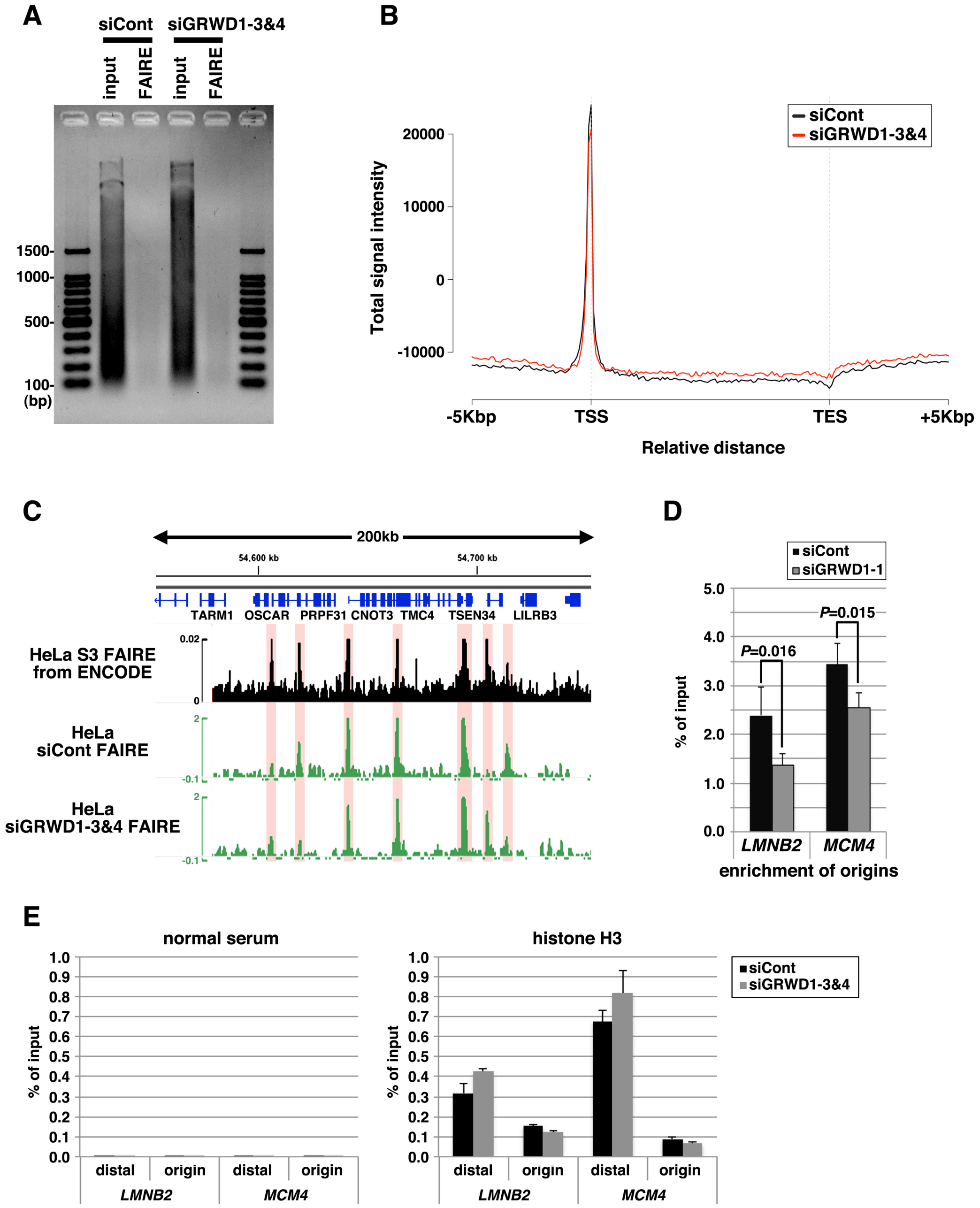

Supplementary Figure S7. Recovery and Analyses of FAIRE Chromatin DNA (Open Chromatin DNA) from Control or GRWD1 siRNA-Treated HeLa Cells

(A) HeLa cells transfected with control (mixture of siLuci&siGFP) or GRWD1 (mixture of siGRWD1-3&4) siRNAs for 48 hrs were cross-linked with formaldehyde, lysed, and then sonicated. For input control samples, prior to phenol-chloroform extractions, the soluble

chromatin was treated with proteinase K and incubated for 6 hrs at 65°C to reverse cross-linking. Chromatin samples were then subjected to two consecutive phenol-chloroform extractions. For FAIRE chromatin samples, cross-linking reversion was performed after phenol-chloroform extractions. Finally, the DNAs were ethanol precipitated and analyzed by agarose gel electrophoresis followed by EtBr staining. The FAIRE DNAs were recovered with similar efficiency both from control and GRWD1 siRNA-treated HeLa cells.

(B) FAIRE signals identified by Seq are significantly enriched at or near TSSs (Transcription start sites) in both control (mixture of siLuci&siGFP) and GRWD1 (siGRWD1-3&4) siRNA-treated HeLa cells. We aligned the FAIRE-Seq signals with gene bodies throughout the genome, revealing that positive signals are enriched around TSSs both in control and GRWD1-silenced cells. Marks -5 kbp and +5 Kbp on the x-axis indicate 5 kb upstream of TSSs and 5 kb downstream of TESs (Transcription end sites), respectively.

(C) Visual representation of FAIRE-Seq results from control (mixture of siLuci&siGFP) or GRWD1 (siGRWD1-3&4) siRNA-transfected HeLa cells for a genomic locus on human chromosome 19 (43). Reads were processed as described in Method. For comparison, previously reported data from HeLa S3 (http://genome.ucsc.edu/ENCODE/downloads.html; wgEncodeOpenChromFaireHelas3Sig.bigWig) are presented in parallel. Representative FAIRE-Seq pattern with sharp peaks at a locus on chromosome 19 (43) was also observed in our assay.

(D) GRWD1 depletion with another siRNA (siGRWD1-1) also reduces chromatin openness at the replication origins at the lamin B2 and MCM4 loci. The FAIRE DNA was analyzed by qPCR using the primers depicted in Figure 2E. Results are shown as percent of input DNA. The means±S.D. are shown (n=3).

(E) GRWD1 depletion with siRNAs does not change the histone H3 protein levels at the lamin B2 and MCM4 replication origins. HeLa cells transfected with control (mixture of siLuci&siGFP) or GRWD1 (siGRWD1-3&4) siRNAs for 48 hr were subjected to ChIP-qPCR with anti-histone H3 antibody that may recognize pan-histone H3. In these assays, cells were cross-linked with 1% formaldehyde for 5 min and sonicated for 18 min (30 sec on/60 sec off cycles). The means±S.D. are shown (n=3).

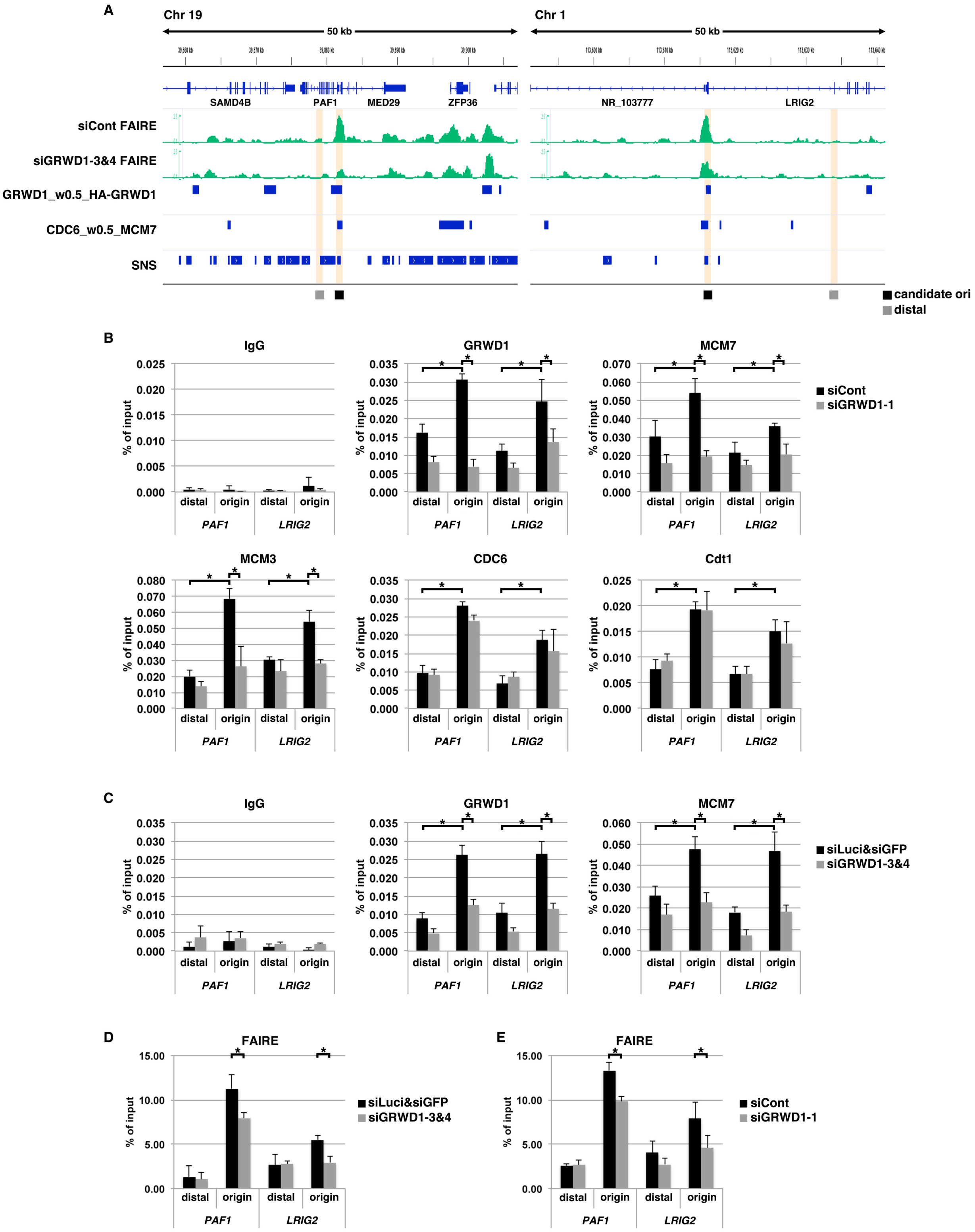
A
Chr 19
50 kb
Chr 1
50 kb
SAMD4B
PAF1
MED29
ZFP36
NR_103777
LRIG2
siCont FAIRE
siGRWD1-3&4 FAIRE
GRWD1_w0.5_HA-GRWD1
CDC6_w0.5_MCM7
SNS
candidate ori
distal
B
IgG
GRWD1
MCM7
MCM3
CDC6
Cdt1
% of input
distal
origin
PAF1
LRIG2
siCont
siGRWD1-1
C
IgG
GRWD1
MCM7
siLuci&siGFP
siGRWD1-3&4
D
FAIRE
siLuci&siGFP
siGRWD1-3&4
E
FAIRE
siCont
siGRWD1-1

Supplementary Figure S8. Pre-RCs Are formed on Two New Candidate Replication Origins in a GRWD1-Stimulated Manner and GRWD1 Affects Chromatin Openness at These Sites

(A) Schematic diagram of the two new candidates of replication origins at the human *PAF1* and *LRIG2* loci. Positions of qPCR primer pairs used to detect the candidate origins and the origin-distal sequences are shown below (black and grey boxes, respectively). qPCR primers were synthesized with the following sequences: PAF1 distal, 5'-AGTGTGCTGAGTACCTGGTTTC-3' and 5'-ATTGCTCGGGAGTACAACTGG-3'; PAF1 ori, 5'-TCGGAACCACACACGGATTG-3' and 5'-CTCTCCACCAATCACAGAAGCA-3'; LRIG2 distal, 5'-TGGAACATCAGCTTGGAATCAC-3' and 5'-GCATCTCTGGATTAAACATCCCAC-3'; LRIG2 ori, 5'-ACTGCAGTCGCAGGAAATTG-3' and 5'-CTCCCCCTCCTTTTGGTCAC-3'. (B) HeLa cells transfected with control or GRWD1 (siGRWD1-1) siRNAs for 48 hr were subjected to ChIP-qPCR with the indicated antibodies and the primers described above. The means±S.D. are shown (n=4). *, $p<0.05$. (C) Silencing of GRWD1 with other siRNAs (siGRWD1-3&4) also inhibits MCM loading at these replication origins. The means±S.D. are shown (n=4). *, $p<0.05$. (D) GRWD1 depletion reduces chromatin openness at these replication origins. Asynchronous HeLa cells were treated with control (mixture of siLuci&siGFP) or GRWD1 (mixture of siGRWD1-3&4) siRNAs for 48 hr and subjected to FAIRE-qPCR using the primers described above. The means±S.D. are shown (n=4). *, $p<0.05$. (E) GRWD1 depletion with another siRNA (siGRWD1-1) also reduces chromatin openness at these replication origins. The means±S.D. are shown (n=4). *, $p<0.05$.

SUPPLEMENTARY MATERIALS AND METHODS

Cell Culture and Synchronization

HEK293T, T98G, HeLa, and HFF2/T (normal human fibroblasts immortalized by telomerase) cells (4) were grown in Dulbecco's modified Eagle's medium with 8% fetal calf serum. Synchronization of T98G cells in the G0, G1/S, and late S/G2/M phases was achieved as described previously (15). To monitor S-phase progression, HeLa cells were synchronized in S phase by treatment with 2.5 mM hydroxyurea (HU) for 18hrs, released, and harvested at the indicated time points. Flow cytometry analysis was performed as described previously (15). Double staining with anti-BrdU antibody and propidium iodide was carried out using BrdU Labeling and detection Kit I (Roche) and Cycletest Plus DNA reagent Kit (BO Biosciences).

Immunoprecipitation, Chromatin Binding Assay, and Immunoblotting

Immunoprecipitation from nuclear extracts and chromatin-binding assay were performed as described previously (15). Immunoblotting and quantification of the band signals were also performed as described previously (15).

For immunoprecipitation-re-immunoprecipitation experiments in Figure 2C, the first immunoprecipitates prepared with anti-Flag antibody (M2, Sigma) were eluted with NET gel buffer containing 3xFlag peptide at 150 μg/ml and then were re-immunoprecipitated with anti-T7 antibody (Novagen).

GST Pull-Down Assay

GST-Cdt1 or GST-CDC6 fusion proteins were bacterially produced and purified as described previously (28, 50). Recombinant GRWD1 was expressed in *E. coli* as a GST-fusion protein and purified on glutathione beads. If necessary, untagged GRWD1 was prepared with PreScission protease (Amersham BioScience), in accordance with the manufacturer's instructions. GST pull-down assay was performed as described previously (15).

Histone Binding and Histone Chaperone Assay

To obtain highly purified recombinant GST-GRWD1-His, the protein was expressed in *E. coli* and purified sequentially on glutathione beads and Ni^{2+}-chelating affinity beads. Core histones were purified from HeLa cells as described previously (51). Preparation of recombinant histone proteins (H2A/H2B and H3-H4 complexes) and a 195 bp DNA fragment containing the *Lytechinus variegatus* 5S ribosomal RNA gene was carried out as described previously (32, 33).

GST pull-down assay to test histone binding was performed essentially as described previously (51), except that bound proteins were eluted directly into SDS sample buffer. Topological assay and nucleosome assembly assay were performed as described previously (32, 33), except that pGEX plasmid was used in the topological assay instead of circular ϕX174 dsDNA.

ChIP-qPCR Assay

ChIP-qPCR assays were carried out as described previously (15).

Transfection and Retrovirus Infection

Expression plasmids (total ~12 μg) were transiently transfected into 3×10^6 HEK293T cells in 100 mm culture dishes using TransIT-293 (Mirus, Madison, WI), in accordance with the manufacturer's instructions, or using polyethylenimine 'MAX' (PEI, Polyscience, Inc. Cat. 24765, Linear) as described previously (52). At 48 (Trans-IT) or 42 (PEI) hrs after transfection, cells were harvested and subjected to immunoprecipitation or immunoblotting analysis.

HeLa cells overexpressing HA-GRWD1 were established by retroviral vectors with pCLMSCVhyg-HA-GRWD1 as described previously (4).

Plasmids

Mammalian expression vectors, pCLMSCVhyg-T7-Cdt1, pCLMSCVhyg-HA-GRWD1, and pcDNA3.1-3HA-Cdt1, and bacterial expression vectors pGEX-6P-1-Cdt1, pGEX-6P-2-GRWD1,

and pGEX-6P-1-CDC6 were produced as described previously (6, 28). pEGFP-C1 was purchased from Clontech. pGEX6P-2-GRWD1 ΔC was created by introducing a stop codon at Phe207 using oligonucleotide-directed mutagenesis (QuikChange Site-directed Mutagenesis Kit; Stratagene). Sequences of the oligonucleotides will be provided upon request. Detailed procedures for construction of pGEX6P-2-GRWD1 Δacid and pGEX6P-2-GRWD1-His will be also provided upon request.

Antibodies

Preparation of polyclonal rabbit antibodies against human GRWD1, Cdt1, ORC1, CDC6, and MCM7 was described previously (6, 30, 50, 53, 54, 55). Specificity of the antibodies has been carefully validated, which is presented in our previous and present papers. Some examples are listed below. For anti-MCM7 antibody, we show specific immunoprecipitation of MCM7 (53, 54). For anti-CDC6 antibody, we confirm the specificity and further show that the signal detected by the antibody is diminished when CDC6 is silenced using siRNAs (28, 55). For anti-Cdt1 antibody, we show that the signal detected by the antibody is diminished when Cdt1 is silenced using siRNAs (Ref. 15 and Supplementary Fig. S2C). For anti-GRWD1 antibody, using siRNA-mediated depletion, we show specific detection of GRWD1 by the antibody in immunoblotting and immunostaining (Supplementary Figure S1A, B). For specificity of these antibodies in ChIP experiments, see Supplementary Figure S5A and its legend.

Other antibodies were purchased from different companies: GST (G7781, Sigma), cyclin A (6E6, Lab Vision), cyclin E (sc-247, Santa Cruz Biotechnology), phospho-histone H3 (number9706, Cell Signaling), ATM (2C1, Gene Tex), Ser1981-phosphorylated ATM (200-301-400, ROCKLAND Biosciences), histone H3 (#07-690, Millipore), HA (3F10, Roche), and actin (MAB1501, CHEMICON).

siRNA Experiments

HeLa cells were transfected with siRNA duplexes using Lipofectamine RNAiMAX (Invitrogen) according to the manufacturer's instructions. In siRNA experiments for GRWD1 silencing, cells were twice transfected with siRNAs.

siRNA oligonucleotides were synthesized (IDT) with the following sequences (sense strand):

siGRWD1-1 (5'-GGGAUGAGCAGGCCCAAAUGAAGdCdC-3'),
siGRWD1-2 (5'-GGGUUCCUCCAACACAAUUUGCUdTdC-3'),
siGRWD1-3 (5'-GGCUCCUGAGCAGCAAUAAAGGAdCdC-3'),
siGRWD1-4 (5'-GACGUAUUGUUCUCUAGAAGGCCdTdG-3'),
siCdt1-1 (5'-GCUGUUGUACUAUCAUGAGCCCUdGdG-3'),
siCdt1-4 (5'-UGGAUGAAGUACCCGACAUCGAGdCdC-3'),
siCdt1-5 (5'-GCACCAGGAGGUCAGAUUACCAGdCdT-3'),
siCDC6-1 (5'-AGACUAUAACUCUACAGAUUGUGdAdA-3'),
siCDC6-3 (5-GGAGGACACUGGUUAAAGAAUUUdAdT-3'),
control DS scrambledNeg (5'-CUUCCUCUCUUUCUCUCCCUUGUdGdA-3'),
control siLuci (5'-GGUUCCUGGAACAAUUGCUUUUAdCdA-3'), and
control siGFP (5'-ACCCUGAAGUUCAUCUGCACCACdCdG-3').

Immunostaining

Cells were fixed with 3.7% formaldehyde in PBS, extracted with 1% Triton X-100 in PBS, and probed with the indicated 1st antibodies. The samples were then incubated with Alexa Fluor 594-conjugated goat anti-mouse IgG antibody and/or Alexa Fluor 488-conjugated goat anti-rabbit IgG antibody (Molecular Probes), counterstained with DAPI, and analyzed with Leica FW4000 and Keyence BZ-9000 microscopes.

Statistical Analysis

Unless otherwise stated, statistical analyses were performed with a two-tailed Student's *t*-test. p values of <0.05 were considered statistically significant.

SUPPLEMENTARY REFERENCES

50. Sugimoto,N., Tatsumi,Y., Tsurumi,T., Matsukage,A., Kiyono,T., Nishitani,H. and Fujita,M. (2004) Cdt1 phosphorylation by cyclin A-dependent kinases negatively regulates its function without affecting geminin binding. *J. Biol. Chem.,* **279,** 19691-19697.
51. Nishimoto,N., Watanabe,M., Watanabe,S., Sugimoto,N., Yugawa,T., Ikura,T., Koiwai,O., Kiyono,T. and Fujita,M. (2012) Heterocomplex formation by Arp4 and beta-actin is involved in the integrity of the Brg1 chromatin remodeling complex. *J. Cell. Sci.,* **125,** 3870-3882.
52. Uno,S. and Masai,H. (2011) Efficient expression and purification of human replication fork-stabilizing factor, claspin, from mammalian cells: DNA-binding activity and novel protein interactions. *Genes Cells,* **16,** 842-856.
53. Fujita,M., Kiyono,T., Hayashi,Y. and Ishibashi,M. (1996) hCDC47, a human member of the MCM family. Dissociation of the nucleus-bound form during S phase. *J. Biol. Chem.,* **271,** 4349-4354.
54. Fujita,M., Kiyono,T., Hayashi,Y. and Ishibashi,M. (1997) In vivo interaction of human MCM heterohexameric complexes with chromatin. Possible involvement of ATP. *J. Biol. Chem.,* **272,** 10928-10935.
55. Fujita,M., Yamada,C., Goto,H., Yokoyama,N., Kuzushima,K., Inagaki,M. and Tsurumi,T. (1999) Cell cycle regulation of human CDC6 protein. Intracellular localization, interaction with the human mcm complex, and CDC2 kinase-mediated hyperphosphorylation. *J. Biol. Chem.,* **274,** 25927-25932.
